# Mitogen-Activated Protein Kinase Cross-Talk Interaction Modulates the Production of Melanins in Aspergillus fumigatus

**DOI:** 10.1128/mBio.00215-19

**Published:** 2019-03-26

**Authors:** Adriana Oliveira Manfiolli, Filipe Silva Siqueira, Thaila Fernanda dos Reis, Patrick Van Dijck, Sanne Schrevens, Sandra Hoefgen, Martin Föge, Maria Straßburger, Leandro José de Assis, Thorsten Heinekamp, Marina Campos Rocha, Slavica Janevska, Axel A. Brakhage, Iran Malavazi, Gustavo H. Goldman, Vito Valiante

**Affiliations:** a Faculdade de Ciências Farmacêuticas de Ribeirão Preto, Universidade de São Paulo, Ribeirão Preto, Brazil; b VIB-KU Leuven Center for Microbiology, Flanders, Belgium; c Laboratory of Molecular Cell Biology, Institute of Botany and Microbiology, KU Leuven, Leuven, Belgium; d Leibniz Research Group Biobricks of Microbial Natural Product Syntheses, Leibniz Institute for Natural Product Research and Infection Biology–Hans Knöll Institute (HKI), Jena, Germany; e Department of Molecular and Applied Microbiology, Hans Knöll Institute (HKI,) Jena, Germany; f Transfer Group Anti-infectives, Hans Knöll Institute (HKI), Jena, Germany; g Departamento de Genética e Evolução, Centro de Ciências Biológicas e da Saúde, Universidade Federal de São Carlos, São Carlos, Brazil; h Friedrich Schiller University, Jena, Germany; Duke University Medical Center

**Keywords:** *Aspergillus fumigatus*, GPCR, MAP kinases, melanin

## Abstract

The pathogenic fungus Aspergillus fumigatus is able to adapt to extremely variable environmental conditions. The A. fumigatus genome contains four genes coding for mitogen-activated protein kinases (MAPKs), which are important regulatory knots involved in diverse cellular responses. From a clinical perspective, MAPK activity has been connected to salvage pathways, which can determine the failure of effective treatment of invasive mycoses using antifungal drugs. Here, we report the characterization of the Saccharomyces cerevisiae Fus3 ortholog in A. fumigatus, designated MpkB. We demonstrate that MpkB is important for conidiation and that its deletion induces a copious increase of dihydroxynaphthalene (DHN)-melanin production. Simultaneous deletion of *mpkB* and *mpkA*, the latter related to maintenance of the cell wall integrity, normalized DHN-melanin production. Localization studies revealed that MpkB translocates into the nuclei when A. fumigatus germlings are exposed to caspofungin stress, and this is dependent on the cross-talk interaction with MpkA. Additionally, DHN-melanin formation was also increased after deletion of genes coding for the Gα protein GpaA and for the G protein-coupled receptor GprM. Yeast two-hybrid and coimmunoprecipitation assays confirmed that GpaA and GprM interact, suggesting their role in the MpkB signaling cascade.

## Abstract (precis)

Aspergillus fumigatus is the most important airborne human pathogenic fungus, causing thousands of deaths per year. Its lethality is due to late and often inaccurate diagnosis and the lack of efficient therapeutics. The failure of efficient prophylaxis and therapy is based on the ability of this pathogen to activate numerous salvage pathways that are capable of overcoming the different drug-derived stresses. A major role in the protection of A. fumigatus is played by melanins. Melanins are cell wall-associated macromolecules classified as virulence determinants. The understanding of the various signaling pathways acting in this organism can be used to elucidate the mechanism beyond melanin production and help to identify ideal drug targets.

## INTRODUCTION

Aspergillus fumigatus is a saprophytic fungus mainly found in the soil and organic debris. This fungus is capable of producing myriads of airborne conidia that can survive in a wide range of environmental circumstances (1). The conidia are normally released into the air and, when inhaled by immunocompromised patients, can cause severe diseases, including invasive aspergillosis (IA). An increase in the incidence of IA has been observed in the last decades, and the mortality attributed to IA infections can reach 90%. IA is a multifactorial disease, and A. fumigatus has several phenotypic characteristics that make it an aggressive opportunistic pathogen (2). Several factors contribute to A. fumigatus virulence, such as production of dihydroxynaphthalene (DHN)-melanin, hypoxia resistance, ability to subtract environmental iron, toxin production, thermotolerance, and distinct surface molecules (3–7).

Mitogen-activated protein kinase (MAPK) pathways are important for the transmission, integration, and amplification of signals and are essential components involved in diverse cellular processes in eukaryotes (8). In fungi, MAPK pathways regulate cellular responses to different kinds of stresses (9–11). The central module of each MAPK signaling pathway consists of three protein kinases: a MAP kinase kinase kinase (MAPKKK), a MAP kinase kinase (MAPKK), and a MAPK. The MAPK cascades are normally triggered by upstream sensors (e.g., receptors) and end with the activation of downstream elements, such as transcriptional regulators (12).

MAPK signaling cascades have been well characterized in yeasts (13–16). In filamentous fungi, their function was mainly assigned to pheromone responses and filamentous growth, osmotic stress, and cell wall integrity. Additionally, it was demonstrated that MAPKs influence several phenotypes relevant for pathogenesis in both human and plant pathogens (9, 11).

A. fumigatus contains four MAPKs: MpkA, which mainly regulates cell wall integrity (17); MpkC and SakA, similar to the Saccharomyces cerevisiae Hog1, which are involved in the response to oxidative stress and in polyalcohol sugar metabolism (18–20); and MpkB, homologous to yeast Fus3, thus far uncharacterized (21). A cross talk interaction between SakA and MpkA has already been observed for the adaptation to the antimycotic drug caspofungin (22, 23). In addition, it was shown that MpkC and SakA cooperate in the responses to different types of stresses in A. fumigatus, including the response to osmotic and oxidative stress, high-temperature adaptation, cell wall damage, and virulence (23).

Previous investigations on Fus3/MpkB orthologs in several phytopathogenic fungi showed their conserved important role in plant infection (11). In parallel, in other important human pathogenic fungi, such as Candida albicans and Cryptococcus neoformans, the deletion of their *FUS3* orthologs mainly caused the loss of mating efficiency and decrease of biofilm formation (24, 25). Of note, in the closely related species Aspergillus nidulans, the *mpkB* deletion not only inhibited sexual crossing, as expected, but also affected the production of relevant secondary metabolites, such as the mycotoxin sterigmatocystin, the antitumor agent terrequinone A, and penicillin (26, 27).

In the present study, we set out to analyze the function of the MpkB regulatory network in A. fumigatus. Deletion of *mpkB* increased sensitivity to the glucan synthase inhibitor caspofungin, suggesting a role in cell wall biosynthesis. Furthermore, we observed an increase of dark pigments during growth in liquid medium that was related to DHN-melanin production. Melanins are a class of dark-brown pigments often associated with the cell wall. Their main role is to protect the organisms from exogenous stressors, thereby contributing to the first line of defense against external hazards (7, 28). A. fumigatus produces two types of melanins: pyomelanin, which derives from the catabolism of tyrosine via the intermediate homogentisate, and DHN-melanin, which is produced as a polyketide derivative and is responsible for the gray-green color of the spores (7). Additionally, DHN-melanin plays a crucial role in conidial immune evasion and, consequently, fungal virulence (29–31). In the A. fumigatus genome, the genes coding for DHN-melanin biosynthetic enzymes, as well as those for pyomelanin, are clustered (32, 33).

Signaling pathways that regulate the expression of melanin biosynthesis genes are barely known. DHN-melanin was found to be regulated by cAMP signaling, and in A. fumigatus, two transcription factors responsible for its regulation were identified: DevR, a basic helix-loop-helix (bHLH) transcription factor, and RlmA, a MADS-box transcription factor actively involved in cell wall integrity (34, 35). The production of pyomelanin was also associated with cell wall stress, highlighting its function as a protective compound (36). Highly important here, we observed that the deletion of the gene coding for the Gα protein GpaA and the gene coding for the G protein-coupled receptor GprM resulted in phenotypes similar to that resulting from *mpkB* deletion, suggesting that they are involved in the same signaling pathway. Here, we present a detailed characterization of the MAPK MpkB in A. fumigatus that led to the identification of its physiological role and several components of its regulatory circuits.

## RESULTS

### MpkB represses DHN-melanin production and conidiation in liquid medium.

**FIG 1:**
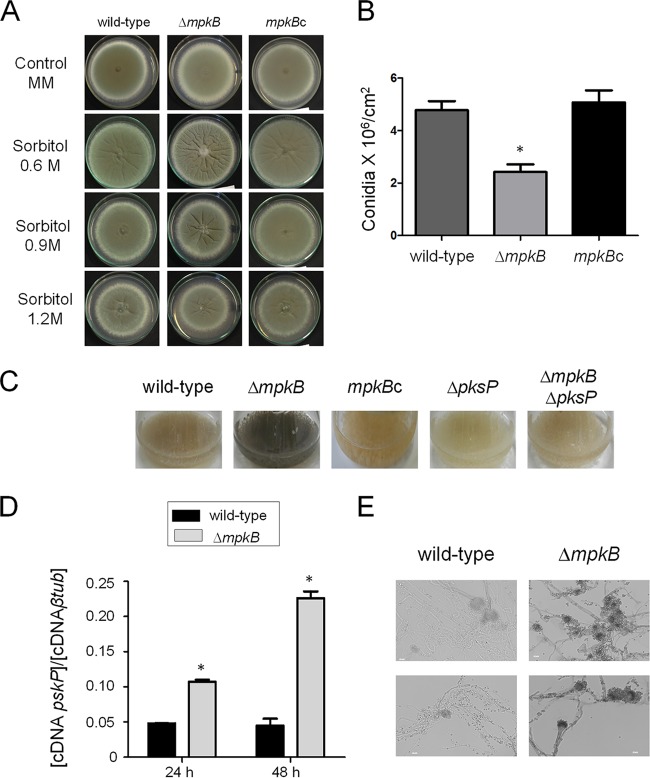
MpkB is important for repressing melanin production. (A) Phenotypic tests were performed by inoculating A. fumigatus conidia onto solid MM agar plates with or without sorbitol. Agar plates were incubated for 5 days at 37°C. (B) Numbers of conidia produced in the wild-type, Δ*mpkB*, and *mpkB*c (complemented) strains. The results are the averages of three repetitions ± standard deviations. One-way analysis of variance (ANOVA) with Tukey’s multiple comparison test was used for statistical analysis, and an asterisk represents a *P* value of <0.05. (C) A. fumigatus conidia were inoculated into liquid MM for 72 h at 37°C with 200 rpm shaking. (D) Results of qRT-PCRs showing the A. fumigatus *pksP* mRNA steady-state levels in the wild-type and Δ*mpkB* strains grown at 37°C for 24 and 48 h, respectively. The results represent the averages of three repetitions ± standard deviations and are expressed as the cDNA concentration of *pksP* divided by the cDNA concentration of the β-tubulin gene. The results are the averages of three repetitions ± standard deviation. One-way ANOVA with Bonferroni posttest was used for statistical analysis, and an asterisk represents a *P* value of <0.05. (E) Visualization of melanin by copper-silver staining of hyphae and conidiophores of wild-type and Δ*mpkB* strains grown for 48 h at 37°C. Bars, 10 µm.

To investigate the function of *mpkB* in A. fumigatus, the complete gene was first deleted and then the mutant strain obtained was complemented by reinsertion of the wild-type gene. The deletion of *mpkB* in A. fumigatus did not affect the radial growth on solid minimal medium (MM) (Fig. 1A); however, a significant reduction in the number of conidia produced by the Δ*mpkB* strain was observed (Fig. 1B). Radial growth assays conducted by challenging the mutant with different exogenous stresses demonstrated that the growth of the Δ*mpkB* strain was not affected by high-temperature, cell wall, and oxidative stresses (Congo red, calcofluor white [CFW], SDS, menadione, paraquat, *t*-butyl hydroxyperoxide, and H_2_O_2_) (Fig. S1 and S2 in the supplemental material). Additionally, when the Δ*mpkB* strain was grown for 48 to 72 h in liquid MM, a dark pigment was produced and released (Fig. 1C). In order to test whether there was an increase of DHN-melanin in the Δ*mpkB* mutant, the *pksP* gene, encoding the polyketide synthase essential for conidial pigment formation and DHN-melanin biosynthesis, was deleted (7). Neither the Δ*pksP* mutant nor the Δ*mpkB* Δ*pksP* double mutant was able to produce the dark color (Fig. 1C). Furthermore, reverse transcription-quantitative PCR (qRT-PCR) analysis of the *pksP* transcription levels revealed increases of 2- to 5-fold in the Δ*mpkB* mutant compared to the levels in the wild type when both strains were grown in liquid MM for 24 and 48 h, respectively (Fig. 1D). Finally, based on the methods reported by Butler et al., we established a protocol for copper-silver staining to directly visualize the melanin produced by the *mpkB* mutant strain (37). As shown by the results in Fig. 1E, silver accumulation was higher in the hyphae, conidiophores, and conidia of the Δ*mpkB* mutant than in the wild-type strain, confirming that the accumulated black pigment was DHN-melanin.

10.1128/mBio.00215-19.4 FIG S1 Growth phenotypes of the wild type and MAPK and complemented mutants grown in the presence of Congo Red (CR) (A), calcofluor white (CFW) (B), and sodium dodecyl sulfate (SDS) (C). The strains were grown for 48 hours at 37°C on MM agar plates. The numbers of conidia are also reported. Download FIG S1, PDF file, 0.2 MB. Copyright © 2019 Manfiolli et al. 2019 Manfiolli et al. This content is distributed under the terms of the Creative Commons Attribution 4.0 International license.

10.1128/mBio.00215-19.5 FIG S2 Growth phenotypes of the wild type and Δ*mpkA*, Δ*mpkB*, and corresponding complemented mutants grown in liquid MM in the presence of *t*-butyl (A), menadione (B), paraquat (C), and hydrogen peroxide (D). The strains were grown for 48 hours at 37°C either on MM agar plates (A to C) or in liquid MM in 96-well plates (D). Numbers of conidia are also reported. Download FIG S2, PDF file, 0.3 MB. Copyright © 2019 Manfiolli et al. 2019 Manfiolli et al. This content is distributed under the terms of the Creative Commons Attribution 4.0 International license.

**FIG 2:**
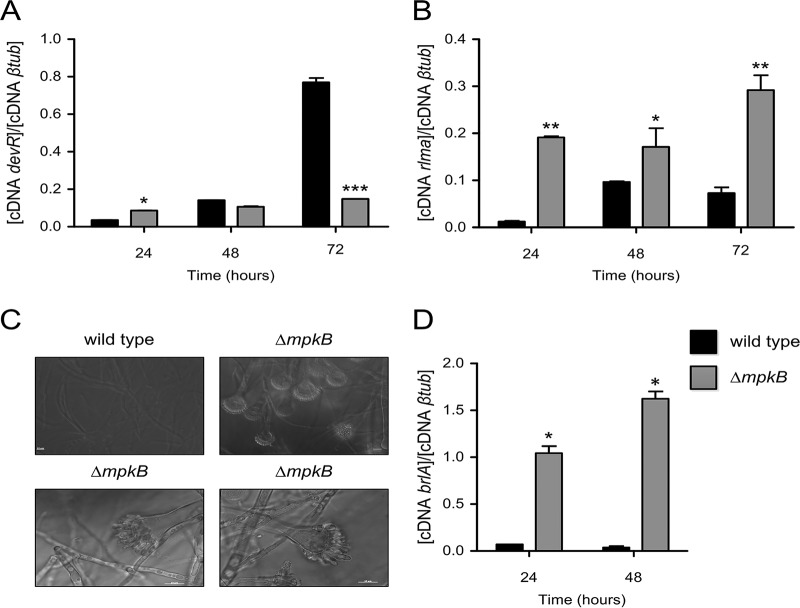
mRNA steady-state levels of the transcription factors of *devR*, *rlmA*, and *brlA* genes. (A, B) Results of qRT-PCRs showing the A. fumigatus *devR* (A) and *rlmA* (B) mRNA accumulation in the wild-type and Δ*mpkB* strains after 24, 48, and 72 h of growth at 37°C. The results are the averages of three repetitions ± standard deviations. One-way ANOVA with Bonferroni posttest was used for statistical analysis. *, *P* < 0.05; **, *P* < 0.01; ***, *P* < 0.001. (C) Hyphae and conidiophores of the wild-type and Δ*mpkB* strains grown for 48 h at 37°C. Bars, 10 µm. (D) *brlA* mRNA steady-state levels detected in the wild-type and Δ*mpkB* strains grown in MM at 37°C for 24 and 48 h, respectively. The results represent the averages of three repetitions ± standard deviations and are expressed as the cDNA concentration of *brlA* divided by the cDNA concentration of the β-tubulin gene; an asterisk represents a *P* value of <0.05.

It was previously reported that two different transcription factors, named DevR and RlmA, regulate the DHN-melanin gene cluster (35). DevR belongs to the bHLH family, while RlmA is a classical MADS-box transcription factor. These two transcription factors regulate the DHN-melanin cluster both positively and negatively, depending on the recognized binding sites on the *pksP* promoter. Additionally, because the assigned binding sites lie close together on the *pksP* promoter region, a parallel binding of the two regulators was already excluded. qRT-PCR analysis showed that the *devR* mRNA steady-state level had doubled in the Δ*mpkB* strain compared to the level in the wild type in the first 24 h of incubation but decreased later on (Fig. 2A). In contrast, the *rlmA* mRNA level was about 10 to 15 times higher in the Δ*mpkB* strain than in the wild type (Fig. 2B). These results suggest that the lack of *mpkB* induced DHN-melanin production by affecting the expression of *rlmA*.

DHN-melanin production is normally associated with conidiation. We also investigated whether the high melanin accumulation was accompanied by deregulated production of conidiophores. Indeed, in the Δ*mpkB* mutant, conidiophores and conidia were produced and released in liquid MM (Fig. 2C). This finding shows that the accumulation of the pigment in the medium was due to the secretion of melanin by the Δ*mpkB* mutant strain.

It was previously reported that the transcription factor BrlA is important in activating the development of conidiation in A. fumigatus (38). Expression studies showed that the *brlA* transcript is expressed 10 to 15 times more in liquid MM in the Δ*mpkB* mutant than in the wild-type strain (Fig. 2D). These results indicate that MpkB plays an important role in the prevention of conidium formation during active vegetative growth.

### Gα protein GpaA and G protein-coupled receptor GprM are important for repressing DHN-melanin production.

**FIG 3:**
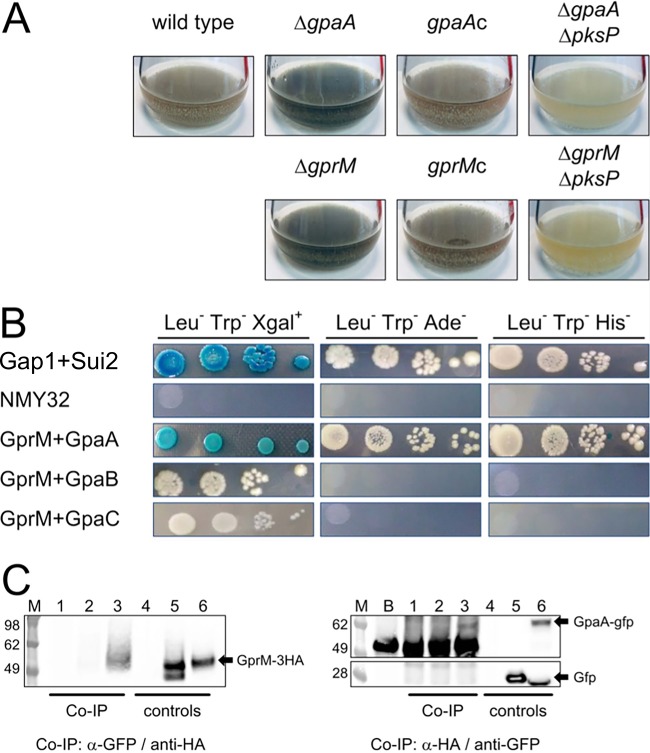
The Gα protein GpaA and the G protein-coupled receptor GprM are important for repressing melanin production. (A) A. fumigatus conidia from the reported strains were inoculated into liquid MM for 72 h at 37°C. (B) Split-ubiquitin yeast two-hybrid screen with A. fumigatus GprM as the bait and the three Gα proteins as preys. Two other yeast proteins, Gap1 and Sui2, previously shown as interacting by using a split-ubiquitin yeast two-hybrid screen, were used as positive controls (41). (C) Coimmunoprecipitation (co-IP) was performed with total protein extracted from the A. fumigatus wild-type strain (lanes 1 and 4), A. fumigatus expressing a dicistronic gene including the *gfp*- and the HA-tagged *gprM* (lanes 2 and 5), and A. fumigatus expressing a dicistronic gene including the *gfp*-tagged *gpaA* and the HA-tagged *gprM* (lanes 3 and 6). Co-IP performed with an anti-GFP antibody specifically isolates the GprM-3×HA protein only in the presence of the GFP-tagged GpaA (left, lane 3). Similarly, co-IP performed using an anti-HA antibody specifically isolates the GpaA-GFP protein, but not GFP alone (right, lane 3). The experiment also shows that the anti-GFP antibody used recognizes an unspecific band detectable also when the beads are not attached to any protein (right, lane B). However, because of the difference in size, a clear band showing the expressed GpaA-GFP protein was detected (right, lane 3). Lanes M, SeeBlue plus2 marker (ThermoFisher).

It has been previously reported that *pksP* transcription is connected to cAMP signaling via protein kinase A (PKA) (34). In order to identify further pathways involved in DHN-melanin regulation, we exploited the dark-color production as a readout system to identify additional signal proteins. Previous studies reported that the deletion of *gpaB*, coding for a Gα protein involved in the cAMP/PKA pathway, strongly decreased DHN-melanin production (39). Besides GpaB, A. fumigatus has two additional Gα proteins, GpaA and GpaC (40). Phenotypic analysis of mutant strains with deletions of the three Gα proteins revealed that only the Δ*gpaA* mutant showed increased pigment production in submerged culture (Fig. 3A). Parallel deletion of the *pksP* gene in the Δ*gpaA* mutant background confirmed that, in this strain also, the dark pigment was DHN-melanin (Fig. 3A).

In eukaryotes, Gα proteins are normally associated with receptors, and in particular G protein-coupled receptors (GPCRs). We screened a comprehensive collection that included 12 A. fumigatus GPCR null mutants (Table 1). Spores for each mutant were inoculated into liquid medium at 37°C for 3 days with shaking (data not shown). Among the screened mutants, only the Δ*gprM* mutant produced more dark pigments than the wild type, and in this case also, Δ*gprM* complementation and the parallel deletion of the *pksP* gene confirmed that the phenotype was connected to DHN-melanin (Fig. 3A).

**TABLE 1:** A. fumigatus strains used in this study

Description	Target gene	Relevant genotype^*a*^	Reference or source
Wild type		CEA10	62
Δ*akuB* (recipient strain)	AFUB_019720	CEA17 *akuB*::*pyrG*; PyrG^+^	63
Δ*mpkA*	AFUB_070630	Δ*akuB mpkA*::*ptrA*; PT^R^	36
*mpkA*c (complemented Δ*mpkA* strain)		Δ*akuB* Δ*mpkA* Δ*mpkA*::*mpkA-hph*; Hyg^R^ PT^R^	36
Δ*mpkB*	AFUB_078810	Δ*akuB mpkB*::*ptrA*; PT^R^	This study
Δ*mpkB*::*mpkB^+^*		Δ*akuB* Δ*mpkB* Δ*mpkB::mpkB-hph*; Hyg^R^	This study
Δ*pksP*	AFUB_033290	Δ*akuB pksP*::*hph*; Hyg^R^	35
Δ*mpkB* Δ*pksP*		Δ*akuB* Δ*mpkB pksP*::*hph*; Hyg^R^ PT^R^	This study
Δ*mpkA* Δ*mpkB*		Δ*akuB* Δ*mpkB mpkA*::*ptrA*; Hyg^R^ PT^R^	This study
Δ*gpaA*	AFUB_012620	Δ*akuB gpaA*::*ptrA*; PT^R^	This study
*gpaA*c		Δ*akuB* Δ*gpaA gpaA-hph*; Hyg^R^ PT^R^	This study
Δ*gpaA* Δ*pksP*		Δ*akuB* Δ*gpaA pksP*::*hph*; Hyg^R^ PT^R^	This study
Δ*gpaB*	AFUB_012410	ATCC 46645 *gpaB*::*hph*; Hyg^R^	39
Δ*gpaC*	AFUB_036760	Δ*akuB gpaC*::*ptrA*; PT^R^	This study
Δ*gprM*	AFUB_090880	Δ*akuB gprM*::*ptrA*; PT^R^	This study
*gprM*c		Δ*akuB* Δ*gprM* Δ*gprM*::*gprM-hph*; Hyg^R^	This study
Δ*gprM* Δ*pksP*		Δ*akuB* Δ*gprM pksP*::*hph*; Hyg^R^ PT^R^	This study
*mpkB-gfp*		Δ*akuB mpkB*::*mpkB-gfp-hph*; Hyg^R^	This study
Δ*mpkA mpkB-gfp*		Δ*akuB mpkB-gfp mpkA*::*ptrA*; Hyg^R^ PT^R^	This study
*gpaA*-*gfp gprM-*3×HA		CEA10 *gpaA*-*gfp gprM-*3×HA; PT^R^	This study
*gfp gprM-*3×HA		CEA10 *gfp gprM-*3×HA; PT^R^	This study
Δ*hmgA*	AFUB_021290	Δ*akuB hmgA*::*ptrA*; PT^R^	32
Δ*hppD*	AFUB_021270	Δ*akuB hppD*::*ptrA*; PT^R^	32
Δ*gprA*	AFUB_034900	Δ*akuB gprA*::*hph*; Hyg^R^	This study
Δ*gprB*	AFUB_055410	Δ*akuB gprB*::*hph*; Hyg^R^	This study
Δ*gprC*	AFUB_090350	Δ*akuB gprC*::*hph*; Hyg^R^	This study
Δ*gprD*	AFUB_028290	Δ*akuB gprD*::*hph*; Hyg^R^	This study
Δ*gprH*	AFUB_052640	Δ*akuB gprH*::*hph*; Hyg^R^	This study
Δ*gprI*	AFUB_047630	Δ*akuB gprI*::*hph*; Hyg^R^	This study
Δ*gprJ*	AFUB_007220	Δ*akuB gprJ*::*hph*; Hyg^R^	This study
Δ*gprK*	AFUB_101830	Δ*akuB gprK*::*hph*; Hyg^R^	This study
Δ*gprO*	AFUB_038590	Δ*akuB gprO*::*hph*; Hyg^R^	This study
Δ*gprP*	AFUB_073100	Δ*akuB gprP*::*hph*; Hyg^R^	This study
Δ*nopA*	AFUB_088000	Δ*akuB nopA*::*hph*; Hyg^R^	This study

a Hyg^R^, hygromycin; PT^R^, pyrithiamine.

In order to check whether the GprM receptor and GpaA interact directly, we employed a split-ubiquitin yeast assay two-hybrid screen with A. fumigatus GprM as bait and the three Gα proteins as preys. This experiment clearly demonstrated that among the Gα proteins expressed by A. fumigatus, GprM interacts exclusively with GpaA (Fig. 3B). Two other yeast proteins, S. cerevisiae Gap1 and Sui2, previously shown to interact by using a split-ubiquitin yeast assay two-hybrid screen, were used as positive controls (41).

Finally, the interaction between the GprM receptor and GpaA was validated in A. fumigatus. We expressed a green fluorescent protein (GFP)-tagged *gpaA* gene with a hemagglutinin (HA)-tagged *gprM* as a dicistronic gene. The two fragments were spaced by the 2A peptide sequence and then placed downstream from a tetracycline-inducible promoter, Tet^ON^, optimized for fungal expression (42, 43). The plasmid obtained was used to transform A. fumigatus strain CEA10 by ectopic integration. By using anti-GFP antibody for the coimmunoprecipitation (co-IP) experiment, we could also isolate the expressed GprM-3×HA receptor (Fig. 3C, left). The opposite experiment, co-IP using the anti-HA antibody, gave the same results, with the GpaA-GFP protein identified by Western blotting (Fig. 3C, right). It is worth noticing that the anti-GFP antibody also recognized unspecific bands (∼50 kDa) from the beads; however, because of the difference in size compared to that of the GpaA-GFP protein, a clear band was still discernible.

### Cross-talk interaction between the MpkB and MpkA pathways.

**FIG 4:**
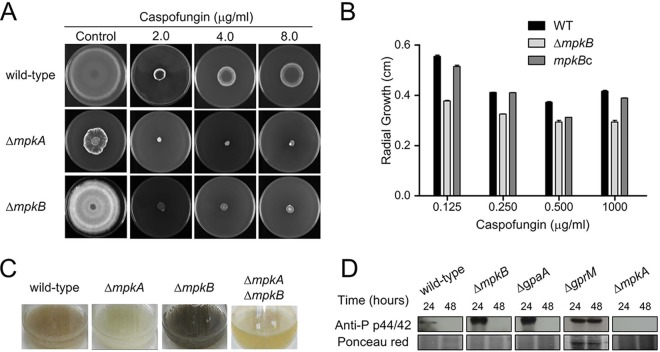
Cross-talk interaction between MpkB and MpkA. (A) The wild-type, Δ*mpkA*, and Δ*mpkB* strains were grown for 5 days at 37°C in the presence of different caspofungin concentrations. (B) Growth defects in the Δ*mpkB* mutant were detected by measuring the radial diameter upon incubation with caspofungin and comparing with those of the wild type and the *mpkB*c complemented strain. Results are expressed as the average radial diameters of three repetitions ± standard deviations. Statistical analysis was performed by using two-way ANOVA with Bonferroni posttest. *, *P* < 0.001. (C) A. fumigatus conidia were inoculated into liquid MM for 72 h at 37°C with shaking at 200 rpm. (D) Immunoblot analyses for MpkA phosphorylation. The wild-type and the mutant strains were grown in MM for 24 and 48 h at 37°C. Anti-p44/42 MpkA antibody was used to detect the phosphorylation levels of MpkA. Ponceau red was used as a control for loading.

The screening conducted against the different cell wall-acting compounds revealed that the Δ*mpkB* mutant is more sensitive to the β-1,3-glucan synthase inhibitor caspofungin, with significant reduction of growth when compared to that of the recipient strain (Fig. 4A and B and Fig. S1). The Δ*mpkB* mutant had also lost the caspofungin paradoxical effect, a phenomenon where high caspofungin concentrations revert the anticipated inhibition of A. fumigatus growth (Fig. 4A and B) (44). This sensitivity was almost comparable with that observed for the Δ*mpkA* mutant; MpkA is the second p42/44 kinase encoded by A. fumigatus and mainly responsible for cell wall maintenance under stress conditions (45). Concerning MpkA, previously published transcriptomic studies reported that this kinase positively affects the expression of the DHN-melanin gene cluster (22, 46). These observations had not yet been proved by *ad hoc* experiments. We constructed an Δ*mpkA* Δ*mpkB* double deletion strain aiming to investigate possible interactions between the two kinases. As shown by the results in Fig. 4C, pigmentation of the medium was not observed for either the Δ*mpkA* or the Δ*mpkA* Δ*mpkB* mutant, suggesting that MpkA positively regulates DHN-melanin biosynthesis while MpkB negatively affects its production.

To investigate whether MpkB repression affects MpkA activation, we checked the MpkA phosphorylation levels in the mutants by immunoblot assay. We detected higher MpkA phosphorylation during the first 24 h of incubation for the Δ*mpkB* and Δ*gpaA* strains than for the wild type, and in the Δ*gprM* mutant, MpkA was shown to be highly activated even 48 h after incubation (Fig. 4D). These data confirmed that GpaA and GprM act upstream from MpkB and that the inhibition of this pathway increased MpkA phosphorylation, leading to higher DHN-melanin accumulation. The mechanism is still unknown; however, an indirect interaction is most likely, since binding between MpkA and MpkB was not detected by a coimmunoprecipitation assay performed with the two kinases (Fig. S3).

10.1128/mBio.00215-19.6 FIG S3 Coimmunoprecipitation assay performed with tagged MpkA and MpkB. (A) Diagnostic PCR to validate the in-locus homologous insertion of the *mpkA*-3×HA construct in the *mpkB-gfp* strain. (B) Phenotypic analysis of wild type, *mpkB-gfp*, and *mpkB-gfp mpkA-*3×HA strains grown on MM agar plates for 4 days at 37°C. (C, D) Affinity purification assays for 3×HA-tagged MpkA and GFP-tagged MpkB were performed with GFP-Trap beads (C) and anti-HA beads (D) to verify interactions. The coimmunoprecipitated proteins were analyzed using the indicated antibodies. The coimmunoprecipitation protocol used is reported in Text S1. Download FIG S3, PDF file, 0.1 MB. Copyright © 2019 Manfiolli et al. 2019 Manfiolli et al. This content is distributed under the terms of the Creative Commons Attribution 4.0 International license.

**FIG 5:**
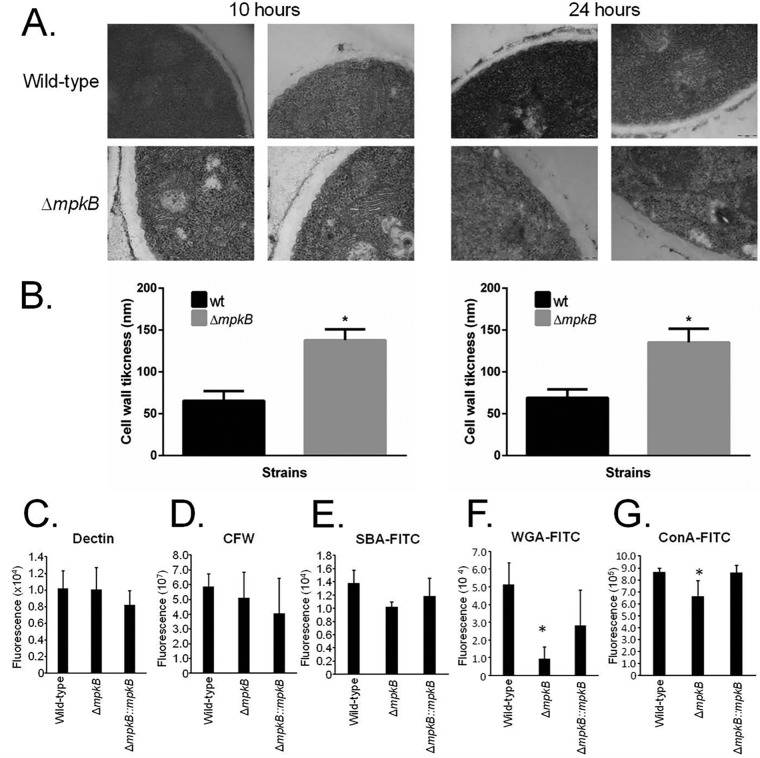
The Δ*mpkB* mutant strain has an altered cell wall organization. (A) Transmission electron microscopy of mycelial sections of the A. fumigatus wild-type and Δ*mpkB* strains grown for 10 h or 24 h in MM at 37°C. (B) The cell wall thicknesses (nm) of 100 sections of different hyphal germlings (average of 4 sections per germling) were measured when grown under the same conditions as specified in the legend to panel A. The averages and standard deviations of the 50 measurements are presented. Statistical analysis was performed using the one-tailed, paired *t* test, comparing data to the results for the control condition (*, *P < *0.00001). (C to G) Detection of different sugars exposed on the cell surface. Conidia were cultured in liquid MM to the hyphal stage, UV killed, and stained with CFW, soluble dectin-1, or specific probe to detect the content of exposed sugars. Experiments were performed in triplicate, and the results are displayed as mean values with standard errors (two-way ANOVA followed by Tukey’s; *, *P < *0.05).

Transmission electron microscopy (TEM) analysis showed that untreated Δ*mpkB* germlings (10 and 24 h) grown in MM had approximately 2-fold-thicker cell walls than the wild type (Fig. 5A and B). Cell wall stains and lectins were used to identify differences in the content or exposure of different carbohydrates on the surface of the fungal cell wall in both the wild type and the Δ*mpkB* mutant. These included the following: (i) soybean agglutinin (SBA)-fluorescein isothiocyanate (FITC) (preferentially binds to oligosaccharide structures with terminal α- or β-linked *N*-acetylgalactosamine [GalNAc] and, to a lesser extent, galactose residues, which are important for recognizing galactosaminogalactan [GAG]); (ii) wheat germ agglutinin (WGA)-FITC (recognizing surface-exposed glucosamine [Glc]); (iii) ConA (concanavalin A)-FITC (recognizes α-linked mannose); (iv) soluble dectin-1 stain (recognizing β-glucans); and (v) CFW (recognizing chitin). There were no differences in the levels of β-1,3-glucan, chitin, or *N*-acetylgalactosamine in the wild-type, Δ*mpkB*, and complemented strains (Fig. 5C to E and G). In contrast, the Δ*mpkB* mutant had about 3-fold-less glucosamine and a lower level of α-linked mannose than the wild-type strain (Fig. 5F).

**FIG 6:**
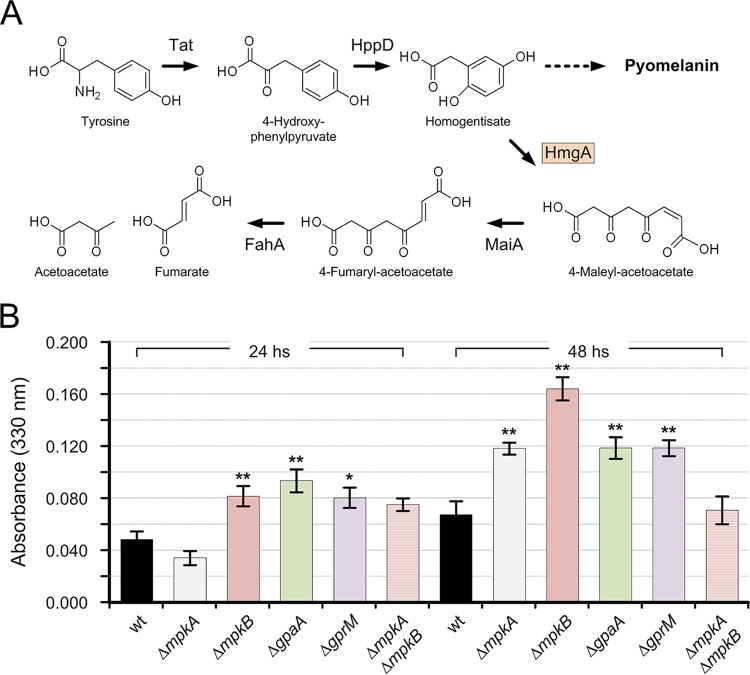
Homogentisate dioxygenase (HmgA) activity assay. (A) Tyrosine degradation pathway showing pyomelanin production due to either an increase or decrease in HgmA (homogentisate dioxygenase) activity (Tat, tyrosine aminotransferase; HppD, 4-hydroxyphenylpyruvate dioxygenase; MaiA, 4-maleylacetoacetate; FahA, fumarylacetoacetate hydrolase). (B) Total proteins extracted from the wild type and Δ*mpkA*, Δ*mpkB*, Δ*gpaA*, Δ*gprM*, and Δ*mpkA* Δ*mpkB* mutant strains were assayed for HmgA activity. After 18 h of growth, 10 mM l-tyrosine was added to the culture. Samples were harvested, and the protein extracts were incubated with homogentisate for 35 min. Formation of maleylacetoacetate was monitored at 330 nm. Total protein extracted from the Δ*hmgA* and Δ*hppD* mutant strains was used as the negative and positive control, respectively (data not shown). HmgA activity was determined in protein extracts of mycelia harvested after 24 and 48 h postinoculation (average value ± standard deviation). Statistical significance was determined for the results of all of the experiments by Student’s *t* test. Significance of differences of data in comparison to the results for the wild-type strain is indicated as follows: *, *P* < 0.1; **, *P* < 0.01.

Finally, we investigated whether the misbalance in MpkA phosphorylation caused any effect on the production of pyomelanin. As previously reported, the cell wall stress induced by the deletion of *mpkA* promotes the formation of pyomelanin as a compensatory response (36). The increase in pyomelanin formation can be detected by testing the activity of the homogentisate 1,2-dioxygenase (HmgA) involved in tyrosine degradation (32). After adding l-tyrosine to the medium, a reduction in HmgA activity induced higher pyomelanin accumulation, and vice versa (Fig. 6A). By growing the mutants in liquid MM plus l-tyrosine for 24 h, we could not detect a significant change in HmgA activity for the *mpkA* deletion mutants. However, impairing MpkB signaling resulted in higher HmgA enzymatic activities for the Δ*gprM*, Δ*gpaA*, and Δ*mpkB* deletion mutants (Fig. 6B). This effect was conserved during the incubation time and was still detectable after 48 h. The mutant with the deletion of *mpkA* showed an increase of HmgA activity after 48 h of incubation, which is not in line with our previous results (36). For comparison to the results of the earlier activity test, we performed the HmgA activity test using smaller volumes (each sample had a total volume of 100 μl and was disposed in a 96-well plate) and reading the absorbance using a plate reader. However, aside from this incongruence, the double deletion of *mpkA* and *mpkB* restored the HmgA activity level, confirming the involvement of the cell wall integrity pathway in pyomelanin formation and highlighting once again the conflicting roles of the MpkA and MpkB pathways.

### Cell wall stress enhances MpkB nuclear localization.

**FIG 7:**
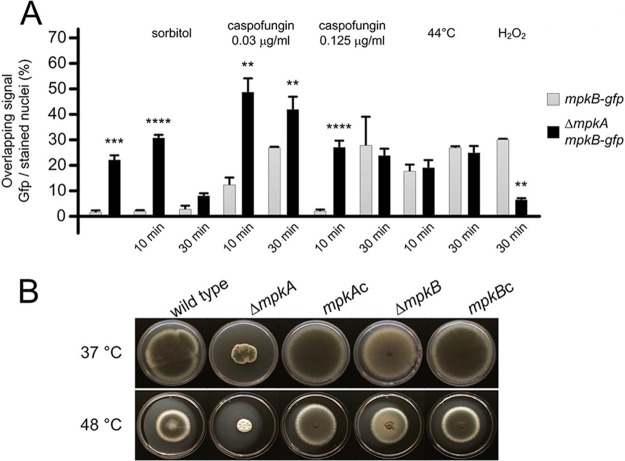
The influence of MpkA on the MpkB-GFP nuclear translocation. (A) Conidia were grown for 16 h at 30°C before stress induction. The compounds used and the times of the induced stresses are also reported. The data represent the averages of three repetitions ± standard deviations. The nuclei of about 50 germlings were counted in each repetition (*, *P* < 0.05; **, *P* < 0.005; ***, *P* < 0.001; ****, *P* < 0.0001). Hoechst counterstaining was used to confirm the nuclear localization of the GFP signal. (B) The wild-type, Δ*mpkA*, and Δ*mpkB* strains were grown on MM for 5 days at 37 and 44°C, respectively.

To assess the involvement and subcellular localization of MpkB during growth and under stress conditions, we generated a strain expressing MpkB-GFP under the control of its own endogenous promoter. This was achieved by replacing the wild-type allele with the *mpkB-gfp* cassette; the strain generated behaved identically to the wild-type strain (Fig. S3). When the MpkB-GFP strain was grown in MM for 16 h at 30°C, a very weak and diffuse fluorescent signal was observed in the cytosol and about 1% of the germlings’ stained nuclei overlapped with the GFP signal (Fig. 7A). The increase of MpkB nuclear localization was observed upon *mpkA* deletion, and this condition was similar when the growing mycelium was challenged with the osmotic stress inducer sorbitol. Moreover, the exposure to different caspofungin concentrations increased nuclear localization over time, with MpkB localized in almost one-third of the germlings 30 min after incubation. As for sorbitol, this phenomenon was strongly enhanced by *mpkA* deletion. These data confirmed that MpkB can activate compensatory pathways in the absence of MpkA during cell wall stress.

Cell wall-impaired mutants are normally sensitive to heat shock (36). For the Δ*mpkB* strain, incubation at high temperatures affected the sporulation ability more than it did radial growth (Fig. 7B). MpkB nuclear localization was increased by incubating A. fumigatus at 44°C (Fig. 7A); however, the parallel deletion of *mpkA* did not affect MpkB activation, demonstrating that the interaction of the two pathways is stress related. This was even clearer when A. fumigatus was challenged with H_2_O_2_. As previously reported, hydrogen peroxide strongly induces MpkA activation, and the Δ*mpkA* mutant is very susceptible to oxidative stress (47). Here, we observed that H_2_O_2_ enhanced MpkB localization to the nucleus as well, but the lack of *mpkA* significantly decreased MpkB nuclear transport. It is possible that an additional salvage pathway that is not activated by MpkB operates during oxidative stress. This observation is supported by the fact that *mpkB* deletion did not affect H_2_O_2_ sensitivity (Fig. S2), suggesting that during oxidative stress, MpkB is transported into the nuclei but is likely still inactive.

### A. fumigatus Δ*mpkB* is still virulent in a low-dose murine infection model.

In order to determine whether the lack of *mpkB* affects A. fumigatus virulence, the Δ*mpkB* strain, the *mpkB*c (complemented) strain, and the respective wild-type strain were tested in a murine infection model of IA (Fig. S4). The results of this experiment indicated that *mpkB* is not essential for virulence of A. fumigatus.

10.1128/mBio.00215-19.7 FIG S4 (A) Survival rates of mice infected with conidia from wild-type, Δ*mpkB*, and *mpkB*c strains. Mice in groups of 10 per strain were infected intranasally with a 20-μl suspension containing 2 × 10^5^ conidia. (B) Histopathology of lung tissue from mice infected with the wild-type, Δ*mpkB*, and *mpkB*c strains plus the PBS control using periodic acid-Schiff (PAS) staining. All infected mice displayed normal fungal persistence compared to the uninfected mice. Download FIG S4, PDF file, 0.2 MB. Copyright © 2019 Manfiolli et al. 2019 Manfiolli et al. This content is distributed under the terms of the Creative Commons Attribution 4.0 International license.

### Ethics statement.

The principles that guide our studies are based on the Declaration of Animal Rights ratified by the UNESCO in January 27, 1978, in its eighth and 14th articles. All protocols used in this study were approved by the local ethics committee for animal experiments from the Campus of Ribeirão Preto, Universidade de São Paulo (Permit Number: 08.1.1277.53.6; Studies on the interaction of A. fumigatus with animals). All animals were housed in groups of five within individually ventilated cages and were cared for in strict accordance with the principles outlined by the Brazilian College of Animal Experimentation (Princípios Eticos na Experimentacão Animal—Colégio Brasileiro de Experimentacão Animal, COBEA) and Guiding Principles for Research Involving Animals and Human Beings, American Physiological Society. All efforts were made to minimize suffering. Animals were clinically monitored at least twice daily and humanely sacrificed if moribund (defined by lethargy, dyspnea, hypothermia, and weight loss). All stressed animals were sacrificed by cervical dislocation.

## DISCUSSION

A number of studies have characterized MAPKs with a focus on their role in pathogenesis and secondary metabolite production (9, 10, 48, 49). The pathogenic fungus A. fumigatus codes for four MAPKs, and until now, MpkB was the only one still uncharacterized. As shown by the experiments whose results are described here, the deletion of the A. fumigatus *mpkB* gene reduced conidium production on solid medium but induced the formation of conidiophores in submerged cultures. In A. nidulans, MpkB was found to be essential for sexual development, and the Δ*mpkB* mutant produced conidia in submerged cultures with constant *brlA* mRNA accumulation (50, 51). We also observed increased accumulation of*brlA* during growth of the A. fumigatus Δ*mpkB* mutant in submerged cultures, indicating that in both fungal systems, MpkB is important for temporal *brlA* expression. However, other than its role in spore development, MpkB had no impact on A. fumigatus virulence, since the Δ*mpkB* mutant showed wild-type-like virulence in a murine infection model. Previously, Atoui et al. (26) have shown that in the A. nidulans Δ*mpkB* mutant, the expression of genes involved in sterigmatocystin, penicillin, and terrequinone A biosynthesis, as well as the expression of *laeA* (loss of aflatoxin expression A, encodes global regulator of secondary metabolism), were significantly reduced (26). Similarly, MpkB also affects secondary metabolite production in A. fumigatus, in particular DHN-melanin, but surprisingly, does not confer resistance to oxidative stress agents. As shown herein, the MpkB-GFP translocation to the nucleus is affected by the presence of MpkA during cell wall stress. The negative interaction between MpkA and MpkB seems to be mutual in particular during cell wall stress; indeed, the deletion of *mpkB* increased MpkA phosphorylation, while the deletion of *mpkA* increased MpkB nuclear localization. Since MpkA phosphorylation was increased in the Δ*mpkB* mutant, the higher melanin production seems to be related to aberrant MpkA activation.

**FIG 8:**
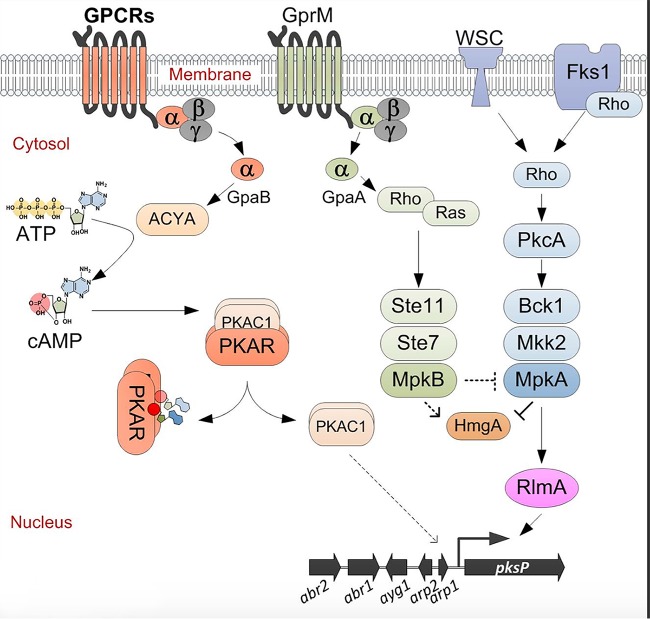
Proposed model for the induction of DHN-melanin biosynthesis. The cAMP pathway activates the melanin cluster via protein kinase catalytic subunit 1 (PkaC1), as previously reported (39, 53). The GPCR GprM, which interacts with GpaA, activates the MpkB pathway. MpkB activation affects MpkA, which regulates the DHN-melanin gene cluster. Finally, the transcription factor RlmA directly regulates the expression of the melanin genes by directly binding the *pksP* promoter (35). Parallel to the expression of the DHN-melanin gene cluster, the activation of MpkB influences the HmgA activity, promoting the accumulation of pyomelanin. ACYA, adenylate cyclase; PKAR, PKA regulatory subunit.

The involvement of the cell wall integrity signaling pathway in melanin production has already been hypothesized. The analysis of transcriptome data of Δ*mpkA* mutants and the involvement of the cell wall-related transcription factor RlmA in the DHN-melanin gene cluster activation already suggested that, besides the cAMP pathway, additional signal pathways are involved in melanin regulation (35, 46, 52). Taking into account that melanin production is lower in the Δ*mpkA* Δ*mpkB* double mutant than in the single *mpkB* deletion strain, this finding supports a model in which the expression of the DHN-melanin biosynthesis genes is controlled by the RlmA transcription factor (Fig. 8).

Thus far, the cAMP/PKA pathway was the only regulatory signaling cascade associated with DHN-melanin production in A. fumigatus (34, 53). Null mutants with deletions of genes involved in this pathway, such as the G protein α subunit GpaB and adenylate cyclase AcyA, showed reductions in DHN-melanin production, while overexpression of the gene encoding the protein kinase A catalytic subunit 1 (PkaC1) induced the expression of the DHN-melanin cluster genes (Fig. 8) (27). This was also observed in other pathogenic fungi, such as C. neoformans, where mutations in the G protein Gpa1, adenylate cyclase Cac1, and/or the catalytic subunit of PKA Pka1 reduced the formation of melanins (54, 55).

Here, we report that an additional G protein α subunit (GpaA) and the GPCR GprM are also involved in the regulation of DHN-melanin production (Fig. 8). Accordingly, GprM physically interacts with GpaA, suggesting that melanin production in A. fumigatus is also mediated via the GprM/GpaA/MpkB pathway. Genome-wide analysis revealed that the A. fumigatus genome codes for three G protein α subunits, one G-β, and one G-γ, while it codes for at least 15 different GPCRs (53, 56). So far, *gprD* and *gprC* have been characterized in A. fumigatus, and their role was connected to hyphal morphogenesis and virulence (57). The high number of GPCRs encoded by fungal genomes makes their study difficult, because the majority of GPCR gene single deletion mutants do not show a discernible phenotype (data not shown). This was also experienced by studying a comprehensive Aspergillus flavus GPCR mutant library, where only a few mutants showed growth defects or impaired toxin production (58). Therefore, until now, there have only been a few examples of filamentous fungi for which the characterization of a signal pathway from the receptor to the activation of response genes has been reported.

In conclusion, here we report the first characterization of MpkB in A. fumigatus, showing its role in conidium formation and development and in the regulation of DHN-melanin production. Additionally, we demonstrate that MpkB interacts with the MpkA signaling pathway, particularly during the caspofungin stress response and melanin production. How this global cross talk affects transcriptional regulators needs to be elucidated, but in the future, the expression of DHN-melanin biosynthesis genes, with its known transcription factors, can be used to address these questions.

## MATERIALS AND METHODS

### Strains and media.

The A. fumigatus strains used in this study are described in Table 1. Detailed information about plasmids and the generation of mutant strains is reported in Text S1 and Fig. S5 in the supplemental material. Complete media were of the following two basic types: a complete yeast extract-glucose (YAG) medium (2% [wt/vol] glucose, 0.5% [wt/vol] yeast extract, 2% [wt/vol] agar, trace elements) with three variants (a YUU medium [YAG medium supplemented with 1.2 g/liter each of uracil and uridine] and liquid YG or YUU medium of the same composition but without agar) and a minimal medium (MM; 1% [wt/vol] glucose, original high nitrate salts, trace elements, 2% [wt/vol] agar, pH 6.5). The trace elements, vitamins, and nitrate salts used were described previously (59). S. cerevisiae strains were grown in synthetic dextrose (SD) medium (6.7 g Difco yeast nitrogen base [YNB] without amino acids, with distilled water to 950 ml, 20 g agar, and 50 ml sterile 40% [wt/vol] glucose).

10.1128/mBio.00215-19.1 TEXT S1 Methods related to the murine infection model, plasmid construction, DNA manipulation, and Southern blot analysis are reported. Download Text S1, PDF file, 0.1 MB. Copyright © 2019 Manfiolli et al. 2019 Manfiolli et al. This content is distributed under the terms of the Creative Commons Attribution 4.0 International license.

10.1128/mBio.00215-19.8 FIG S5 Graphical representation of the mutated loci for the *A. fumigatus* transformant strains analyzed and the respective Southern blots. The genes of interest are shown in blue, their flanking genes are grey. The cassettes for resistance to pyrithiamine (*ptrA*) and hygromycin (*hph*) are labeled in green and pink, respectively. The probes used for Southern blot analyses are marked as red stripes. The DNA ladder used is indicated (Hyperladder 1 kb; Bioline). (A) The *mpkB* gene was deleted, and the mutant strain complemented with the native gene by ectopic integration. (B) The Δ*mpkB* strain was complemented by homologous recombination with the native *mpkB* gene fused to a *gfp* sequence (in yellow) and an *nos* terminator sequence (in black). (C) Deletion of the *mpkA* gene in the *mpkB-gfp* background and in the Δ*mpkB* strain. Southern blots were performed separately. The probe also anneals to an additional ∼2-kb band. (D) Deletion and complementation of the *gprM* locus; the complementation was obtained by homologous recombination. (E) Deletion and complementation of the *gpaA* locus. The complementation was performed by ectopic integration of the *gpaA* wild-type gene. (F) Disruption of the *gpaC* gene. (G) Disruption of the *pksP* gene in the wild-type, Δ*mpkB*, Δ*gpaA*, and Δ*gprM* strains. (H) The dicistronic genes including the *gpaA-gfp gprM*-3×HA and the *gfp gprM*-3×HA sequences were ectopically integrated into the A. fumigatus CEA10 genome. Positive transformants were validated by Western blot analysis using anti-GFP antibodies (left) and anti-HA antibodies (right). The predicted molecular weights of the gene products are also reported. Download FIG S5, PDF file, 0.4 MB. Copyright © 2019 Manfiolli et al. 2019 Manfiolli et al. This content is distributed under the terms of the Creative Commons Attribution 4.0 International license.

### Conidium production.

Five microliters of 2 × 10^7^ conidia of each strain was overlaid onto MM agar plates, followed by incubation at 37°C for 5 days. Fresh conidia were harvested in 10 ml of 0.01% (vol/vol) Tween 80, vortexed for 30 s, and filtered through miracloth. Conidial suspensions were spun for 5 min at 3,000 × *g*, and pellets were resuspended using 10 ml H_2_O. The numbers of conidia were counted using a hemacytometer. This experiment was performed in triplicate.

### Phenotypic assays.

The phenotypes of the deletion mutants were evaluated either by measuring radial growth or by assessing the initial growth of a droplet of conidia from a serial dilution at different temperatures and in the presence or absence of oxidative and osmotic stressing agents. Dropout experiments were performed using 5-µl amounts of a 10-fold dilution series starting at a concentration of 2 × 10^7^ for the wild-type and mutant strains spotted on different growth media and grown for 48 h at 37°C. The A. fumigatus crystal violet assay was performed as previously described (60, 61). All the experiments were performed at least three times. We also screened a collection of 12 GPCR mutants (Table 1) for the production of dark pigments by growing them in liquid MM for 48 h at 37°C.

### Split-ubiquitin yeast two-hybrid assay.

The A. fumigatus *gprM*, *gpaA*, *gpaB*, and *gpaC* open reading frames were synthesized and cloned into the pTMBV4 and pDL2XN plasmids, respectively (41). The genes of interest were expressed in Saccharomyces cerevisiae strain NMY32. The NMY32 strain was initially transformed with the *gprM*-bearing plasmid and later with one of the *gpaA-*, *gpaB-*, or *gpaC*-bearing plasmids. Then, we used these transformants for the split-ubiquitin membrane-based yeast two-hybrid assay. First, these strains were inoculated onto SD-Leu-Trp selective culture medium and grown overnight. The absorbance of the inoculum was measured through spectrophotometry, and later, all samples were standardized to an optical density at 600 nm (OD_600_) of 1. Tenfold serial dilutions were made, and these dilutions were spotted on agar plates with different selective culture media, including SD-Leu-Trp plus X-Gal (5-bromo-4-chloro-3-indolyl-β-d-galactopyranoside), SD-Leu-Trp-Ade, SD-Leu-Trp-His, SD-Leu-Trp-His plus 3-AT (3-amino-1,2,4-triazole), or SD-Leu-Trp-Ade-His medium. The agar plates were incubated for 3 days at 30°C.

### Staining and microscopy.

The copper-silver staining experiments were performed by a modification of a previously described procedure (37). Briefly, the wild-type and Δ*mpkB* mutant strains were grown on solid MM on slides. After growth, the slide cultures were incubated in 10 mM copper sulfate solution at room temperature for 1 h and, after washing, treated with 1.0% (wt/vol) sodium sulfide solution for 1 h in the dark. Subsequently, the slide cultures were incubated for 4 min in a solution of 22 mg silver lactate and 170 mg hydroquinone (HQ) in a citrate buffer (0.1 M, pH 3.7) solution (Ag-HQ solution) at room temperature. The copper and sulfide treatments were followed by repeated washings (six times with 2 ml distilled water). Afterward, slides were visualized on a Zeiss Axio Observer Z1 fluorescence microscope. Bright-field images were captured with an AxioCam camera (Carl Zeiss) and processed using AxioVision software (version 4.8). For fluorescence experiments, MpkB-GFP strain conidia were cultivated on coverslips in 4 ml of YG medium for 16 h at 30°C. After incubation, subsets of coverslips with adherent germlings were left untreated or treated with 1.0 M sorbitol, different concentrations of H_2_O_2_, or 0.125 µg/ml caspofungin for different periods of time. Subsequently, the coverslips were rinsed with phosphate-buffered saline (PBS; 140 mM NaCl, 2 mM KCl, 10 mM NaHPO_4_, 1.8 mM KH_2_PO_4_, pH 7.4) and incubated for 3 min in a solution with Hoechst stain (Life Technologies) (12 μg/ml). After incubation with the dye, the coverslips were washed with PBS and mounted for examination. Slides were visualized on an Observer Z1 fluorescence microscope using a 100× objective oil immersion lens (for GFP, filter set 38 high efficiency [HE], excitation wavelengths of 450 to 490 nm, and emission wavelengths of 525/50 nm; for Hoechst stain, filter set 49, excitation wavelengths of 365 nm, and emission wavelengths of 420 to 470 nm). Differential interference contrast (DIC) images and fluorescence images were captured with an AxioCam camera and processed using AxioVision software (version 4.8).

### Homogentisate dioxygenase assay.

A. fumigatus was grown in liquid MM at 37°C and 180 rpm shaking for 18 h. Afterwards, 10 mM l-tyrosine was added. Samples for the homogentisate dioxygenase assay were collected 6 and 30 h after induction (in total, 24 and 48 h of growth, respectively). The mycelia were harvested, washed with water, frozen in liquid nitrogen, and stored at −80°C overnight. For protein preparation, the mycelia were powdered in liquid nitrogen and resuspended in 50 mM KPO_4_, pH 7.0. After centrifugation at 16,000 × *g*, 4°C, for 15 min, the supernatants were collected and the protein concentrations were determined using the Coomassie plus protein assay (ThermoFisher Scientific) according to the manufacturer’s instructions. The homogentisate dioxygenase assay was performed as described previously (32), in a 9-well plate in a total volume of 100 µl with 2 µg of total protein for each sample. All measurements were done with three biological and three technical replicates. Proteins extracted from the A. fumigatus Δ*hmgA* and Δ*hppD* mutants were used as negative and positive controls, respectively (not shown) (32). All the measurements were performed using a ClarioSTAR plate reader (BMG Labtech).

### Immunoblot analysis.

To assess the phosphorylation status of MpkA, freshly harvested conidia (1 × 10^7^) of the wild-type and mutant strains were inoculated into 50 ml liquid MM at 37°C for 16 h with 180 rpm. Mycelia were ground in liquid nitrogen with pestle and mortar. Protein extraction was performed as previously described (36). Briefly, 0.5 ml lysis buffer containing 10% (vol/vol) glycerol, 50 mM Tris–HCl, pH 7.5, 1% (vol/vol) Triton X-100, 150 mM NaCl, 0.1% (wt/vol) SDS, 5 mM EDTA, 50 mM sodium fluoride, 5 mM sodium pyrophosphate, 50 mM β-glycerophosphate, 5 mM sodium orthovanadate, 1 mM phenylmethylsulfonyl fluoride (PMSF), and 1× cOmplete mini-protease inhibitor (Roche Applied Science) were added to the ground mycelium. Extracts were centrifuged at 20,000 × *g* for 40 min at 4°C. The supernatants were collected, and the protein concentrations were determined using the Bradford assay (Bio-Rad). Fifty micrograms of protein from each sample was resolved in a 12% (wt/vol) SDS–PAGE gel and transferred to polyvinylidene difluoride (PVDF) membranes (Merck Millipore). The phosphorylation state of MpkA was examined using anti-phospho-p44/42 MAPK antibody (Cell Signaling Technologies) following the manufacturer’s instructions using a 1:1,000 dilution in TBST buffer (137 mM NaCl, 20 mM Tris, 0.1% [vol/vol] Tween 20). Primary antibody was detected using a horseradish peroxidase (HRP)-conjugated secondary antibody raised in rabbit (Sigma). Chemoluminescent detection was achieved using the SuperSignal west pico chemiluminescent substrate (Thermo Scientific).

### Coimmunoprecipitation of GpaA and GprM.

For co-IP studies of GpaA and GprM, C-terminally tagged GpaA-GFP and GprM-3×HA and GFP alone were constructed and *gpaA*-GFP::*gprM*-3×HA and GFP::*gprM*-3×HA strains generated. Amounts of 50 ml of MM were inoculated with 1 × 10^7^ spores of the two mutant strains and the wild type and incubated at 37°C with shaking. After 16 h, 20 µg/ml doxycycline was added. Mycelia were harvested after an additional 4 h of incubation, flash-frozen, and stored at −80°C. For protein preparation, the mycelia were powdered in liquid nitrogen and resuspended in 500 µl Tris buffer (0.5 M NaCl, 0.1 M Tris, pH 8.0) including EDTA-free protease inhibitor cocktail (Roche), 1 mM AEBSF [4-(2-aminoethyl)benzenesulfonyl fluoride hydrochloride], and 1 mM EDTA. Samples were vortexed 3× for 10 s and afterwards centrifuged for 20 min at 4°C and 16,000 × g. The supernatant was removed, and protein concentration was determined using the Bradford assay (Pierce Coomassie-plus; Fisher Scientific). For reciprocal co-IP, protein A Dynabeads (Thermofisher Scientific) were incubated with anti-3×HA (Sigma) or anti-GFP B2 (SantaCruz Biotechnology) antibody for 30 min at room temperature. Before adding 100 µg of protein solution to 80 µl of beads for 3 h while shaking at 8°C, the beads were washed three times with resuspension buffer. After incubation, the beads were washed three times with resuspension buffer and resuspended in 20 µl buffer, and bound proteins were eluted by adding sample buffer and boiling at 95°C for 5 min. Afterwards, proteins were separated on X10 Bolt 4 to 12% Bis-Tris plus gels (Fisher Scientific) and transferred to a nitrocellulose membrane using an iBlot2 blotting system (ThermoFisher Scientific). GFP and GFP-tagged GpaA were detected using an HRP-linked anti-GFP B2 antibody (Santa Cruz Biotechnology) diluted 1:2,500 in blocking solution (5% [wt/vol] milk powder in TBST). 3×HA-tagged GprM was detected using the antibodies and dilutions described above. The antibodies were diluted in blocking solution. For controls for the anti-GFP antibody blotting, 20 µg of wild-type and GpaA-GFP::GprM-3×HA and 1 µg of GFP::GprM-3×HA protein preparations were used. For controls for the anti-3×HA antibody blotting, 2 µg of all protein preparations were used. Detection was performed using WesternSure premium chemiluminescent substrate (Li-Cor GmbH) and a Fusion system (Vilber Lourmat).

10.1128/mBio.00215-19.2 TABLE S1 Primers used in this study. Download Table S1, PDF file, 0.1 MB. Copyright © 2019 Manfiolli et al. 2019 Manfiolli et al. This content is distributed under the terms of the Creative Commons Attribution 4.0 International license.

10.1128/mBio.00215-19.3 TABLE S2 Plasmids used in this study. Download Table S2, PDF file, 0.1 MB. Copyright © 2019 Manfiolli et al. 2019 Manfiolli et al. This content is distributed under the terms of the Creative Commons Attribution 4.0 International license.
