# Supplementary material for: Mitogen-Activated Protein Kinase Cross-Talk Interaction Modulates the Production of Melanins in Aspergillus fumigatus
Source: mBio. 2019 Mar 26;10(2):e00215-19. doi: 10.1128/mBio.00215-19 (PMC6437049; doi:10.1128/mBio.00215-19)
Supplement: FIG S1 [file mBio.00215-19-sf001.pdf]

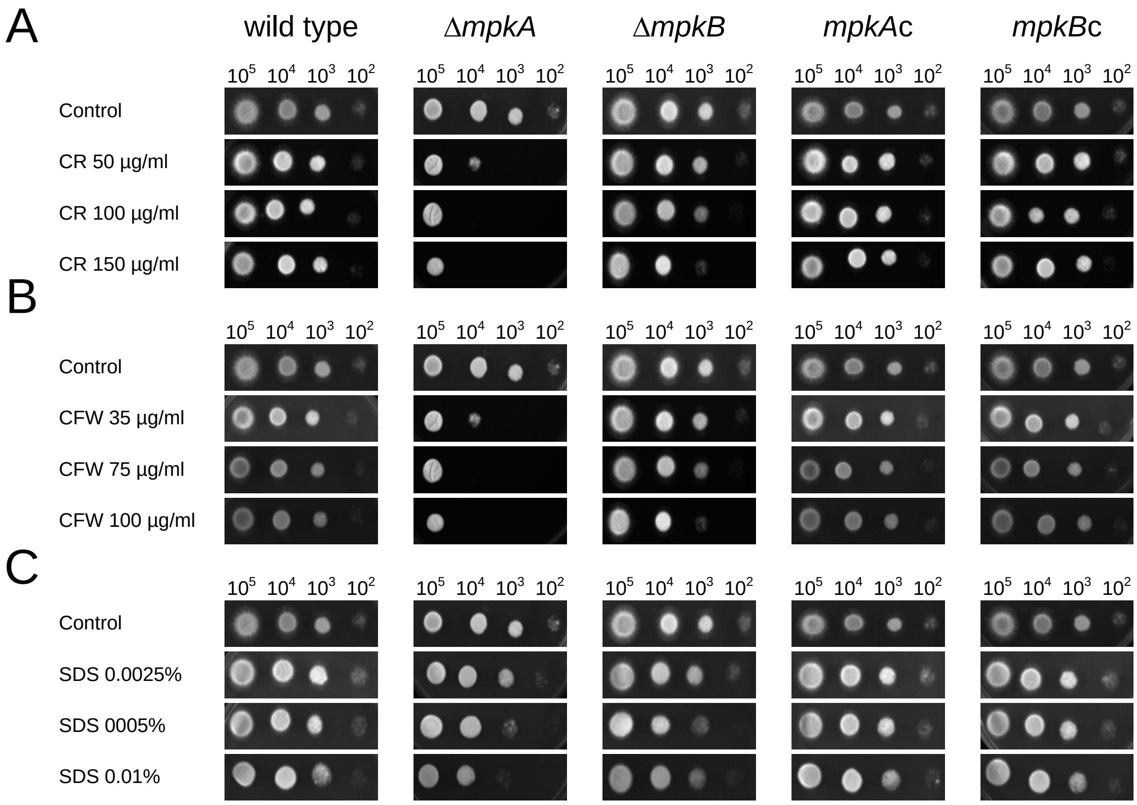

Figure S1. Growth phenotypes of the wild-type, MAPK and complementing mutants grown in presence of (A) Congo Red (CR), (B) Calcoflour White (CFW) and (C) sodium dodecyl sulfate (SDS). The strains were grown for 48 hours at 37 ^{o}C on AMM solid media. The numbers of inoculated conidia are also reported.
