# Supplementary material for: Mitogen-Activated Protein Kinase Cross-Talk Interaction Modulates the Production of Melanins in Aspergillus fumigatus
Source: mBio. 2019 Mar 26;10(2):e00215-19. doi: 10.1128/mBio.00215-19 (PMC6437049; doi:10.1128/mBio.00215-19)
Supplement: FIG S2 [file mBio.00215-19-sf002.pdf]

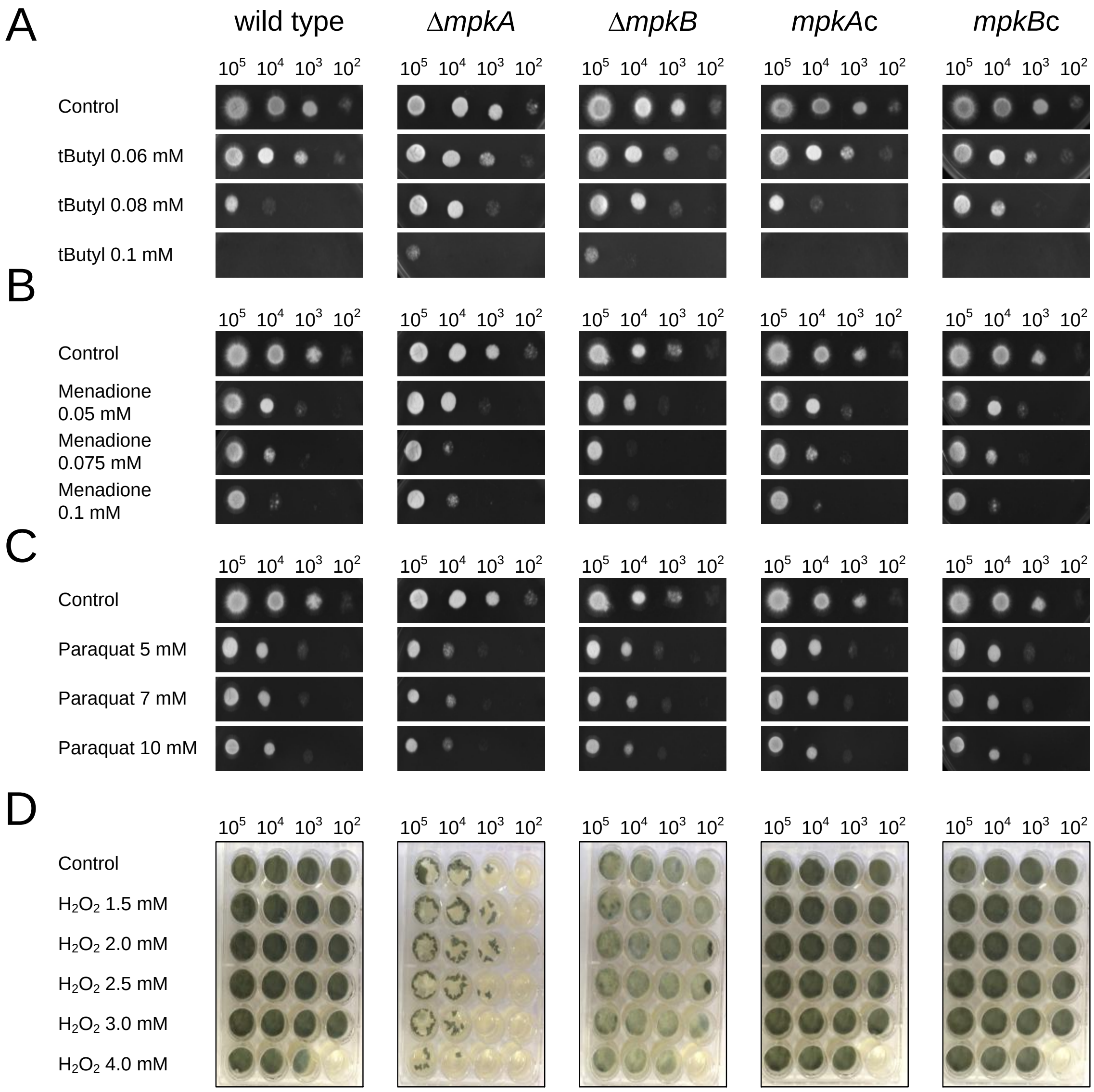

Figure S2. Growth phenotypes of the wild-type, Δ*mpkA*, Δ*mpkB* and corresponding complementing mutants grown in MM in presence of (A) *t*-Butyl, (B) menadione, (C) paraquat, and (D) hydrogen peroxide. The strains were grown for 48 hours at 37 ^{o}C either on solid AMM media (A-C) or on AMM liquid media in 96-wells plates (D). Numbers of inoculated conidia are also reported.
