# Supplementary material for: Mitogen-Activated Protein Kinase Cross-Talk Interaction Modulates the Production of Melanins in Aspergillus fumigatus
Source: mBio. 2019 Mar 26;10(2):e00215-19. doi: 10.1128/mBio.00215-19 (PMC6437049; doi:10.1128/mBio.00215-19)
Supplement: FIG S3 [file mBio.00215-19-sf003.pdf]

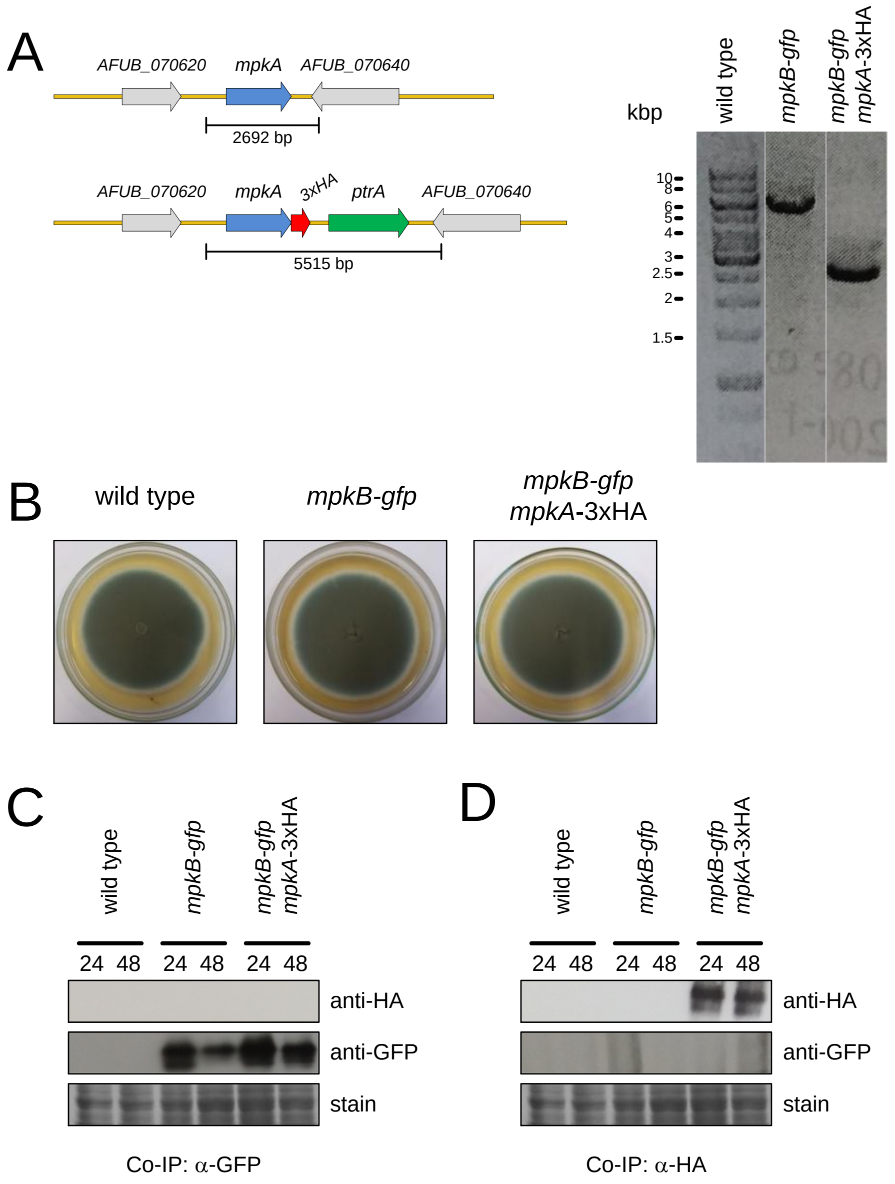

Figure S3. Co-immunoprecipitation assay performed with tagged MpkA and MpkB. (A) Diagnostic PCR to validate the in locus homologous insertion of the *mpkA*-3xHA construct in the *mpkB-gfp* strain. (B) Phenotype analysis of wild type, *mpkB-gfp* and *mpkB-gfp*/*mpkA-3xHA* strains grown on MM agar plates for 4 days at 37 ^{o}C. (C-D) Affinity purification assays for the 3xHA-tagged MpkA and the GFP-tagged MpkB were performed with GFP-Trap beads (C) and anti-HA beads (D) to verify interactions. The co-immunoprecipitated proteins were analyzed by the indicated antibodies. The used co-immunoprecipitation protocol is reported in the supplementary text.
