# Supplementary material for: Mitogen-Activated Protein Kinase Cross-Talk Interaction Modulates the Production of Melanins in Aspergillus fumigatus
Source: mBio. 2019 Mar 26;10(2):e00215-19. doi: 10.1128/mBio.00215-19 (PMC6437049; doi:10.1128/mBio.00215-19)
Supplement: FIG S4 [file mBio.00215-19-sf004.pdf]

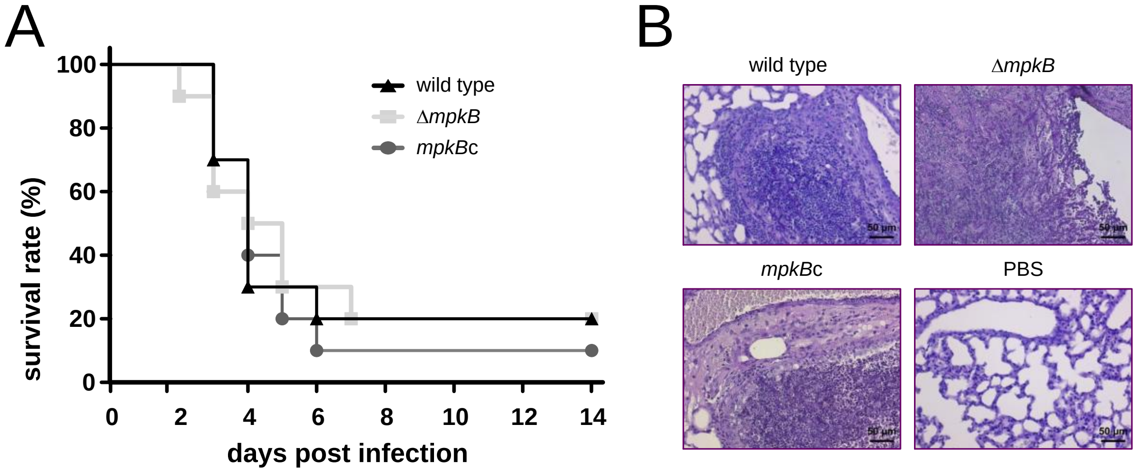

Figure S4. (A) Survival rate of mice infected with conidia from wild-type, Δ*mpkB* and *mpkB*c strains. Mice in groups of 10 per strain were infected intranasally with a 20 µl suspension of conidia at a dose of $2x10^5$. (B) Histopathology of lung tissue from mice infected with the wild-type, Δ*mpkB*, and the *mpkB*c strains plus the PBS-control using PAS staining. All the infected mice displayed a normal fungal persistence when compared to the uninfected ones.
