# Supplementary material for: Mitogen-Activated Protein Kinase Cross-Talk Interaction Modulates the Production of Melanins in Aspergillus fumigatus
Source: mBio. 2019 Mar 26;10(2):e00215-19. doi: 10.1128/mBio.00215-19 (PMC6437049; doi:10.1128/mBio.00215-19)
Supplement: TEXT S1 [file mBio.00215-19-s0001.pdf]

Supplementary information for

Mitogen activated protein kinase cross-talk interaction modulates the production of melanins in *Aspergillus fumigatus*

Adriana Oliveira Manfiolli, Filipe Silva Siqueira, Thaila Fernanda dos Reis, Patrick Van Dijck, Sanne Schrevens, Sandra Hoefgen, Martin Föge, Maria Straßburger, Leandro José de Assis, Thorsten Heinekamp, Marina Campos Rocha, Slavica Janevska, Axel A. Brakhage, Iran Malavazi, Gustavo H. Goldman, and Vito Valiante

Co-immunoprecipitation MpkA and MpkB

To perform co-immunoprecipitation (co-IP) assays, C-terminal HA-tagged MpkA strains were generated in the MpkB::GFP background. The wild type, MpkB-GFP and MpkA-3xHA::MpkB-GFP strains were grown for 24 h and 48 h in MM supplemented with 1% (w/v) glucose (Supplementary Figure S1) or the strains were grown for 16 h in MM before caspofungin was added to a final concentration of 0, 0.2, 2 µg/ml for 1 h. To perform reciprocal co-IP assays, the C-terminal HA-tagged MpkA strain was used. GFP-Trap co-IP experiments were performed as previously reported (4).Briefly, mycelia were frozen with liquid nitrogen, ground, and 500 mg were resuspended in 1 mL of B250 buffer (5). Samples were centrifuged at maximum speed for 10 min at 4 °C. The supernatant was removed, and a Bradford assay (BioRad) was carried out to measure protein content. The same amount of protein for each sample was added to 20 µL of Dynabeads Protein A (Thermo Fisher Scientific) previously incubated with monoclonal anti-HA antibody (Sigma). The resin was washed three times with resuspension buffer prior to incubation. Cell extracts and resin were then incubated under shaking at 4 °C for 2 h. After incubation, the resin was washed three times in resuspension buffer by placing the tube in a DynaMag™ magnet. To release the proteins from the resin, samples were incubated with Sample Buffer and boiled at 98 °C for 5 min. Proteins were transferred from a 10% SDS-PAGE gel onto a nitrocellulose membrane for a Western blot assay using a Trans-Blot turbo transfer system (Bio-Rad). GFP-

tagged MpkB was detected using a rabbit anti-GFP antibody (Abcam) at 1:2,000 dilution and a goat anti-rabbit IgG horseradish peroxidase (HRP) antibody (Cell Signaling Technology) at 1:10,000 dilution. For the HA-tagged MpkA, a mouse monoclonal anti-HA antibody (Sigma) was used at 1:2,000 dilution as a primary antibody followed by an anti-mouse IgG HRP conjugate (Cell Signaling Technology) used at 1:10,000 dilution as a secondary antibody.

Murine infection model. Specific pathogen-free female outbreed CD-1 mice (18-20 g, 8-10 week-old) were supplied by Charles River, Germany. Animals were housed under standard conditions in individually ventilated cages und fed with normal mouse chow and water *ad libitum*. All animals were cared for in accordance with the European animal welfare regulation and approved by the responsible federal/state authority and ethics committee in accordance with the German animal welfare act (permit no. 03-001/12).

Mice were immunosuppressed with two single doses of 25 mg cortisone acetate (Sigma-Aldrich), which were injected intraperitoneally three days before and immediately prior to infection with conidia (day 0). Mice were anesthetized by an intraperitoneal anesthetic combination of midazolam, fentanyl, and medetomidine. $2x10^5$ conidia in 20 µL PBS were applied to the nares of the mice. Anesthesia was terminated by subcutaneous injection of flumazenil, naloxon and atipamezol. Infected animals were monitored twice daily and humanely sacrificed if moribund.

Plasmid construction

All cloning strategies were performed by either classical cloning, employing the plasmids pCR2.1 and pUC19, or transformation-associated recombination (TAR) cloning in the yeast *Saccharomyces cerevisiae*, employing the plasmid pYes2 (1). The used primers are listed in Table S1, while the used plasmids are listed in Table S2. All *A. fumigatus* genes were isolated from the strain CEA17 (2).

The entire *mpkB* gene (AFUB_078810), including 1 kb 5’ and 3’ flanking regions, was isolated using primers [1] and [2], and cloned into pCR2.1, obtaining the plasmid pCR2.1-*mpkB*. This plasmid was subsequently amplified using primers [3] and [4] and re-ligated, obtaining the plasmid pCR2.1-Δ*mpkB*,

owning the *mpkB* flanking regions spaced by a *Sma*I restriction site. The hygromycin (*hph*) resistance cassette was obtained from the plasmid pUC-*hph* digested with *Eco*RV and *Sma*I, while the pyrithiamine (*ptrA*) resistance cassette was obtained from the plasmid pSK275, firstly digested with *Nco*I and *Hin*dIII, and then filled-in. Both fragments were cloned into the linearized vector pCR2.1-Δ*mpkB*, obtaining plasmids pCR2.1-Δ*mpkB::hph* and pCR2.1-Δ*mpkB::ptrA*, respectively. The *A. fumigatus* mutant having *mpkB* deleted using the *ptrA* resistance cassette was complemented by ectopic integration of the plasmid pCR2.1-*mpkB*c, obtained by insertion of the blunt digested *hph* cassette into the vector pCR2.1-*mpkB* previously linearized with *Eco*RV.

The construct *mpkB-gfp* was obtained by TAR cloning by fusing four PCR products into the pYes2 plasmid. The *mpkB* gene, including its native promoter, was amplified using primers [5] and [6], while its 3' flanking region was amplified using primers [7] and [8]. The *gfp* sequence was amplified from plasmid p123 and the *hph* cassette from plasmid pUC-*hph* using primers [9][10] and [11][12], respectively. All fragments were mixed with the linearized pYes2 backbone, obtaining the plasmid pYes2-*mpkB-gfp*. The linearized plasmid was used to complement the *A. fumigatus* mutant having *mpkB* deleted using the *ptrA* cassette.

The *mpkA* gene was deleted by amplification of the *mpkA* deletion cassette with primers [13] and [14] from the plasmid pCR2.1-Δ*mpkA::ptrA*.

The *pksP* gene (AFUB_033290) was amplified using primers [15] and [16], and the fragment was cloned into the pCR2.1 vector. The obtained plasmid pCR2.1-*pksP* was digested with *Hin*cII, and the blunt cut *hph* cassette was cloned to interrupt the native *pksP* gene. The obtained plasmid, having the *hph* cassette flanked by the interrupted *pksP* gene, was designated pCR2.1-Δ*pksP::hph.*

For *gpaA* deletion, the entire gene (AFUB_012620), including both 1 kb flanking regions, was amplified using primers [17] and [18] and cloned into the pCR2.1 vector to yield the plasmid pCR2.1-*gpaA*. The obtained plasmid was digested using the blunt cut enzyme *Bst*Z17I, which led to the removal of the *gpaA* ORF, with the exception of the last 78 bp. The *ptrA* resistance cassette was cut from the pSK275 plasmid using the blunt cut enzymes *Sma*I and *Sci*I, and then

cloned into the digested pCR2.1-*gpaA* vector, giving a deletion plasmid named as pCR2.1-Δ*gpaA::ptrA*. The plasmid used for the complementation of the *A. fumigatus* Δ*gpaA* strain was obtained by amplifying the *gpaA* gene, plus its flanking regions, using the primers [19] and [20], and after *Xba*I digestion, the fragment was cloned in an *Xba*I linearized pUC-*hph* plasmid, yielding pUC-*hph-gpaA*.

The *gpaC* gene (AFUB_036760), including its 1 kb 3' and 5' flanking regions, was amplified using the primers [21] and [22], and cloned into a pCR2.1 vector. The obtained plasmid pCR2.1-*gpaC* was digested with *Nde*I and *Pml*I in order to disrupt the *gpaC* ORF. The digested *Nde*I-*Sma*I *ptrA* cassette was cloned into the linearized pCR2.1-*gpaC*, giving the plasmid pCR2.1-Δ*gpaC::ptrA*.

The deletion cassettes to create the GPCR mutant library were obtained by three-primer-PCR. The primers used to amplify the *gprM* (AFUB_090880) deletion cassette were the pairs [23] [24] and [25] [26] for the flanking regions, and the pair [27] [28] for the *ptrA* cassette isolated from the plasmid pSK275. For the complementation of the Δ*gprM* mutant, the entire *gprM* gene, including its 1 kb promoter region, and the *gprM* 3' 1 kb flanking region were amplified using the primer pairs [29] [30] and [31] [32], respectively. The two fragments were fused to a DNA fragment including the *hph* resistance cassette and the *nosT* terminator amplified from plasmid pYes2-*mpkB-gfp* using the primers [33] and [11]. The three fragments were fused using TAR cloning, obtaining the plasmid pYes2-*gprM*c.

To generate the *mpkA::3xHA::ptrA* fusion fragment, a 1.7 kb portion of DNA consisting of the *mpkA* ORF and 5' upstream region, along with a 1 kb 3' flanking region, were amplified with primer pairs [34] [35] and [36] [37], respectively. The 0.8 kb *3xHA-trpC* fusion was amplified with primers [38] and [39] from the plasmid prs426*sdsA*::*3xHA*. These flanking regions and a *ptrA* fragment amplified from the plasmid pPTRI, were fused into pUC19 using the Gibson Assembly Master Mix (New England Biolabs), resulting in plasmid pUC19*mpkA::3xHA*. This cassette was then transformed into the *mpkB-gfp* strain and verification of MpkA tagging was confirmed *via* PCR (Supplementary Figure S3).

For the cloning of the pTet::*gpaA-gfp*::*gprM-3xHA* and pTet::*gfp*::*gprM-3xHA* co-IP vectors, first a pYes2 derivative was generated harboring the Tet^{ON} inducible promoter as well as the *ptrA* cassette. The cassette was amplified with primer pairs [40][41], while the Tet^{on} promoter was amplified with [42][43] from the template pSH013 (3). Next, *gpaA-gfp* and *gprM-3xHA-troT* fusions were generated. To this end, the plasmid pYes2::*gpaA-gfp* was initially made to tag the *gpaA* locus through homologous recombination. Afterwards, we used this plasmid as template for the expression of the *gpaA*/*gprM* dicistronic gene. The *gpaA* ORF was amplified using primers [44][45], while the *gpaA* 1 kb 3' flanking region was amplified with [46][47]. The obtained fragments were fused to the pYes2 vector with a *gfp-nosT* segment amplified from the plasmid p123 using primers [9][48], and the *ptrA* resistance cassette obtained from the plasmid pSK275 with primers [49][50]. Finally, the co-IP vectors were assembled by TAR cloning. In order to achieve a simultaneous expression of the genes as a dicistrononic gene under the control of a single inducible promoter, the 2A sequence from picornaviruses was inserted between the tagged *gpaA* and *gprM* (3). The pTet::*ptrA* backbone was linearized by PCR using primers [51][52], while the 2A sequence was generated *via* annealing of the two complementary primers [53][54]. The genes of interest were amplified with the following primer pairs: [55][56] for *gpaA-gfp*, [57][58] for *gprM-3xHA* and [59][56] for the *gfp* control.

DNA manipulation and Southern blot analysis

Circular plasmid DNA from yeast and genomic DNA from *A. fumigatus* were extracted using the Master Pure Yeast DNA Purification Kit (Epicentre Biotechnologies, USA).

Southern blot analyses were performed by separating DNA fragments on 1% (w/v) agarose gels and blotting onto Hybond N+ nylon membranes (GE Healthcare Bio-Sciences, Germany). Detection of hybridized probes was performed using the DIG High Prime Labelling and Detection System (GE Healthcare Biosciences, Germany) according to the manufacturer's instructions. Blocking and antibody binding were performed with DIG Easy Hyb (GE Healthcare Biosciences, Germany). Detection was carried out using CDP Star ready-to-use (Roche Diagnostics, Germany) according to the manufacturer's

instructions. The Southern blot results are shown in the Supplementary Figure S5.

References

1. **Kouprina N, Larionov V.** 2008. Selective isolation of genomic loci from complex genomes by transformation-associated recombination cloning in the yeast Saccharomyces cerevisiae. Nat Protoc **3:**371-377.
2. **da Silva Ferreira ME, Kress MR, Savoldi M, Goldman MH, Hartl A, Heinekamp T, Brakhage AA, Goldman GH.** 2006. The akuB(KU80) mutant deficient for nonhomologous end joining is a powerful tool for analyzing pathogenicity in Aspergillus fumigatus. Eukaryot Cell **5:**207-211.
3. **Hoefgen S, Lin J, Fricke J, Stroe MC, Mattern DJ, Kufs JE, Hortschansky P, Brakhage AA, Hoffmeister D, Valiante V.** 2018. Facile assembly and fluorescence-based screening method for heterologous expression of biosynthetic pathways in fungi. Metab Eng **48:**44-51.
4. **Ries LN, Beattie SR, Espeso EA, Cramer RA, Goldman GH.** 2016. Diverse Regulation of the CreA Carbon Catabolite Repressor in Aspergillus nidulans. Genetics **203:**335-352.
5. **Bayram O, Bayram OS, Valerius O, Johnk B, Braus GH.** 2012. Identification of protein complexes from filamentous fungi with tandem affinity purification. Methods Mol Biol **944:**191-205.
6. **Aichinger C, Hansson K, Eichhorn H, Lessing F, Mannhaupt G, Mewes W, Kahmann R.** 2003. Identification of plant-regulated genes in Ustilago maydis by enhancer-trapping mutagenesis. Mol Genet Genomics **270:**303-314.
7. **Valiante V, Jain R, Heinekamp T, Brakhage AA.** 2009. The MpkA MAP kinase module regulates cell wall integrity signaling and pyomelanin formation in Aspergillus fumigatus. Fungal Genet Biol **46:**909-918.
8. **Szewczyk E, Krappmann S.** 2010. Conserved regulators of mating are essential for Aspergillus fumigatus cleistothecium formation. Eukaryot Cell **9:**774-783.
9. **Liebmann B, Muller M, Braun A, Brakhage AA.** 2004. The cyclic AMP-dependent protein kinase a network regulates development and virulence in Aspergillus fumigatus. Infect Immun **72:**5193-5203.
10. **Valiante V, Baldin C, Hortschansky P, Jain R, Thywissen A, Strassburger M, Shelest E, Heinekamp T, Brakhage AA.** 2016. The Aspergillus fumigatus conidial melanin production is regulated by the bifunctional bHLH DevR and MADS-box RlmA transcription factors. Mol Microbiol **102:**321-335.
11. **Manfiolli AO, de Castro PA, Dos Reis TF, Dolan S, Doyle S, Jones G, Riano Pachon DM, Ulas M, Noble LM, Mattern DJ, Brakhage AA, Valiante V, Silva-Rocha R, Bayram O, Goldman GH.** 2017. Aspergillus fumigatus protein phosphatase PpzA is involved in iron assimilation, secondary metabolite production, and virulence. Cell Microbiol **19**.
