# Supplementary material for: Mitogen-Activated Protein Kinase Cross-Talk Interaction Modulates the Production of Melanins in Aspergillus fumigatus
Source: mBio. 2019 Mar 26;10(2):e00215-19. doi: 10.1128/mBio.00215-19 (PMC6437049; doi:10.1128/mBio.00215-19)
Supplement: FIG S5 [file mBio.00215-19-sf005.pdf]

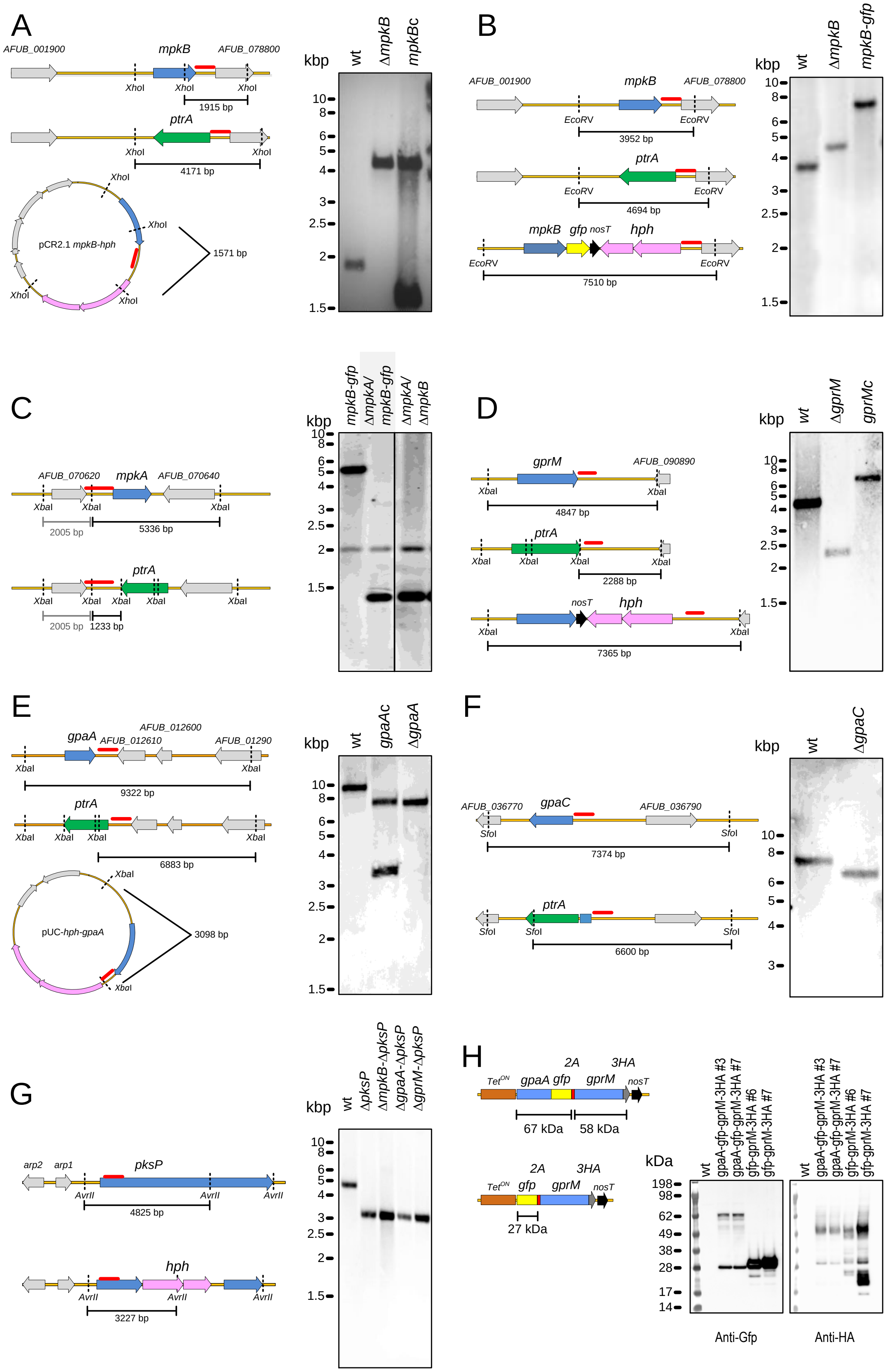
A
AFUB_001900
mpkB
AFUB_078800
XhoI
1915 bp
ptrA
4171 bp
pCR2.1 mpkB-hph
1571 bp
kbp
wt
ΔmpkB
mpkBc
10
8
6
5
4
3
2.5
2
1.5
B
AFUB_001900
mpkB
AFUB_078800
EcoRV
3952 bp
ptrA
4694 bp
mpkB gfp nosT hph
7510 bp
kbp
wt
ΔmpkB
mpkB-gfp
C
AFUB_070620
mpkA
AFUB_070640
XbaI
2005 bp
5336 bp
ptrA
1233 bp
kbp
mpkB-gfp
ΔmpkA/ mpkB-gfp
ΔmpkA/ ΔmpkB
D
gprM
AFUB_090890
XbaI
4847 bp
ptrA
2288 bp
nosT
hph
7365 bp
kbp
wt
ΔgprM
gprMc
E
gpaA
AFUB_012600
AFUB_012610
AFUB_01290
XbaI
9322 bp
ptrA
6883 bp
pUC-hph-gpaA
3098 bp
kbp
wt
gpaAc
ΔgpaA
F
AFUB_036770
gpaC
AFUB_036790
SfoI
7374 bp
ptrA
6600 bp
kbp
wt
ΔgpaC
G
arp2
arp1
pksP
AvrII
4825 bp
hph
3227 bp
kbp
wt
ΔpksP
ΔmpkB-ΔpksP
ΔgpaA-ΔpksP
ΔgprM-ΔpksP
H
Tet^ON
gpaA gfp
2A
gprM
3HA
nosT
67 kDa
58 kDa
gfp
27 kDa
kDa
198
98
62
49
38
28
17
14
wt
gpaA-gfp-gprM-3HA #3
gpaA-gfp-gprM-3HA #7
gfp-gprM-3HA #6
gfp-gprM-3HA #7
Anti-Gfp
Anti-HA

Figure S5. Graphical representation of the mutated loci for the analyzed *A. fumigatus* mutants and their respective Southern blots. The genes of interest are shown in blue, their flanking genes are in gray. The cassettes for resistance to pyrithiamine (*ptrA*) and hygromycin (*hph*) are labeled in green and pink, respectively. The probes used for Southern blot analyses are marked as red stripes. The DNA ladder used is indicated (Hyperladder 1Kb, Bioline). (A) The *mpkB* gene was deleted, and the mutant strain complemented with the native gene by ectopic integration. (B) The Δ*mpkB* strain was complemented by homologous recombination with the native *mpkB* gene fused with a *gfp* sequence (in yellow), and a *nos* terminator sequence (in black). (C) Deletion of the *mpkA* gene in the *mpkB-gfp* background and in the Δ*mpkB* strain. The Southern blots were performed separately. The probe also anneals an additional ~2 Kb band. (D) Deletion and complementation of the *gprM* locus; the complementation was obtained by homologous recombination. (E) Deletion and complementation of the *gpaA* locus. The complementation was performed by ectopic integration of the *gpaA* wild-type gene. (F) Disruption of the *gpaC* gene. (G) Disruption of the *pksP* gene in the wild-type, the Δ*mpkB*, the Δ*gpaA* and the Δ*gprM* strains. (H) The dicistronic genes including the gpaA-gfp/gprM-3HA and the gfp/gprM-3HA sequences were ectopically integrated in the *A. fumigatus* CEA10 strain. Positive transformants have been validated by western-blot using anti-GFP antibodies (left panel) and anti-HA antibodies (right panel). The predicted molecular weights of the gene products are also reported.
