# Supplementary material for: Mitogen-Activated Protein Kinase Cross-Talk Interaction Modulates the Production of Melanins in Aspergillus fumigatus
Source: mBio. 2019 Mar 26;10(2):e00215-19. doi: 10.1128/mBio.00215-19 (PMC6437049; doi:10.1128/mBio.00215-19)
Supplement: TABLE S1 [file mBio.00215-19-st001.pdf]

Supplementary Table S1. Primers used in this study.

N°	Name	Sequence 5'-3'	Usage
1	mpkB_for	CACGATACTATTCGACCTTCC	*mpkB* deletion plasmid
2	mpkB_rev	GACAGGAGTATTTGGCTCTGG	*mpkB* deletion plasmid
3	mpkB_Del_for	GGGAGATTCAGTGGCGTCTCTTG	*mpkB* deletion plasmid
4	mpkB_Del_rev	GGGTTGCTAACTTTCAGCATC	*mpkB* deletion plasmid
5	mpkB_pYes_P11	GCTGTAATACGACTCACTATAGGGAATATTGT TACTAGTGGATCCCCGGGG	*mpkB-gfp* plasmid
6	mpkB_pYes_P2	CGATCCTCCTTGAGGAGGTTGCTGCACCATGG TTGCTAACTTTCAGCATC	*mpkB-gfp* plasmid
7	mpkB_pYes_P3	ATAGTGCCACGTTCTAAATTCAACCAAGGGCG AATTCTGCAGATGGGAG	*mpkB-gfp* plasmid
8	mpkB_pYes_P4	CATAACTAATTACATGATGCGGCCCTCTAGGA CAGGAGTATTTGGCTCTGG	*mpkB-gfp* plasmid
9	eGFP_for_vv	ATGGTGAGCAAGGGCGAGGAGC	*mpkB-gfp* plasmid
10	nosT_hphR_vv	CCCAGCACTCGTCCGAGGGCAAAGGAATAGAT CGATGAATTCTCATGTTTG	*mpkB-gfp* plasmid
11	gpda_for_vv	CTTGGTTGAATTTAGAACGTGG	*mpkB-gfp* plasmid
12	Hph_Rev_vv	TTCCTTTGCCCTCGGACGAGTGC	*mpkB-gfp* plasmid
13	mpkA_for	CTCATTCCTTGTTCTGATGCG	*mpkA* deletion
14	mpkA_rev	GACTGTCGCAGAAATCCGCTTC	*mpkA* deletion
15	pksP_75_for	CAACCTCTTGCAGGCGAAGAAC	*pksP* deletion
16	pksP_-4_rev	GGAACTCATGGCCGTGGCCATG	*pksP* deletion
17	gpaA_for	CATTTCGTCTCGTTCTCCGAAC	*gpaA* deletion plasmid
18	gpaA_rev	CTGTGCCCACTGCCATATCG	*gpaA* deletion plasmid
19	gpaA_XbaI_for	TCTAGAGCTCCTCTTACTGTGTACTC	*gpaA* deletion plasmid
20	gpaA_XbaI_rev	TCTAGACAGATAACCCGCTGCACATG	*gpaA* deletion plasmid
21	gpaC_F_XbaI	CTCCTTGCTCTAGAGGACGC	*gpaC* deletion plasmid
22	gpaC_R_XbaI	CTGAGCTTCTAGACATGCAGAC	*gpaC* deletion plasmid
23	gprM_for	ATTGTCACTCCATGTTCACTCC	*gprM* deletion
24	gprM_rev_ptrA	GGCCTGAGTGGCCATCGAATTCCTTGTTCGTG TGACTTCTGCG	*gprM* deletion
25	gprM_for_ptrA	GAGGCCATCTAGGCCATCAAGCGCTATGTCAT ACCGTTGAGGC	*gprM* deletion
26	gprM_rev	GTGATAGTGCCATGCTGATGC	*gprM* deletion
27	ptrA_for	GAATTCGATGGCCACTCAGGCC	*ptrA* cassette amplification
28	ptrA_rev	GCTTGATGGCCTAGATGGCCTC	*ptrA* cassette amplification
29	gprMc1	GCTGTAATACGACTCACTATAGGGAATATTGT CGGTAGTCTAGACTGTGG	*gprM* complementation
30	gprMc2	GTTTGAACGATCTGCAGCCGGGCGGCCGCTCT AGTCATCATATTCCCGATAG	*gprM* complementation
31	gprMc3	CAATAGTGCCACGTTCTAAATTCAACCAAGCC ACTAGATCCCCGCATTTC	*gprM* complementation
32	gprMc4	CATAACTAATTACATGATGCGGCCCTCTAGGT GAGTCTTCTCTAGACCAC	*gprM* complementation
33	NosT_f	CATAACTAATTACATGATGCGGCCCTCTAGGT GAGTCTTCTCTAGACCAC	*gprM* complementation
34	pUC19 5`ORF mpkA-for	TTGTAAAACGACGGCCAGTGCGGCTACACACG ACACTC	*mpkA-3xHA* plasmid
35	5`ORF mpkA linker 3xHA-rev	TACCACCTCCTTGGACATCCATCCCCCG	*mpkA-3xHA* plasmid

Supplementary Table S1. Primers used in this study (*continued*).

N°	Name	Sequence 5'-3'	Usage
36	ptrA 3`mpkA-for	CCCATGATAGACGTTGACTTTCGTATGAAG	*mpkA-3xHA* plasmid
37	pUC19 3`mpkA-rv	ATCCCCGGGTACCGAGCTCGGAAATCCGCTTC AACACC	*mpkA-3xHA* plasmid
38	linker3xHA trpC-fr	GGAGGTGGTAGCGGTGGT	*mpkA-3xHA* plasmid
39	linker3xHA trpC-rv	CTGTCAGATCTGTAAAAAAGTTTCGGCCGGC	*mpkA-3xHA* plasmid
40	pYes2_ptrA_F	GGGCGCTACAGGGCGCGTGGGGATGATCCAGG GAACAAAAGCTGGGTACC	GpaA-GprM co-IP
41	pYes2_ptrA_R	GGCCTTTCCGGTTTCATACACCGGGCAAAGGG GCTGCAGGAATTCGATGG	GpaA-GprM co-IP
42	pYes2_Tet_F	CTTTGCCCGGTGTATGAAACC	GpaA-GprM co-IP
43	pYes2_Tet_R	CCGCCAGTGTGATGGATATCTGCAGAATTCTG TGATGTGATGGAGTTGAG	GpaA-GprM co-IP
44	gpaAG1	GCTGTAATACGACTCACTATAGGGAATATTGA CTTTCGTTAAGAGACTCC	*gpaA-gfp* plasmid
45	gpaAG2	GGTGAACAGCTCCTCGCCCTTGCTCACCATGA TCAGACCACAGAGTCTG	*gpaA-gfp* plasmid
46	gpaAG3	ACGAGCGTCAGCGCGAGTGTGCTGAGTAAGAG CCATTATACTTGACGGAG	*gpaA-gfp* plasmid
47	gpaAG4	CATAACTAATTACATGATGCGGCCCTCTAGGC GAGGCTGAATTCGAGATG	*gpaA-gfp* plasmid
48	nosT-pyrP	CCTGAGTGGCCATCGAATTCCTGCAGCCCCGA TGAATTCTCATGTTTGAC	*gpaA-gfp* plasmid
49	ptrA_for_2	GGGCTGCAGGAATTCGATGG	*gpaA-gfp* plasmid
50	ptrA-rev3	TTACTCAGCACACTCGCGCTG	*gpaA-gfp* plasmid
51	pTet_ptrA_F	GGCCCTGCATTAATGAATCGG	GpaA-GprM co-IP
52	pTet_ptrA_R	TGTGATGTGATGGAGTTGAGATGGA	GpaA-GprM co-IP
53	2A_F	GGCTCTGGCGCCACCAACTTCTCTCTCCTCAA ACAGGCTGGCGACGTTGAAGAAAACCCTGGCC CT	GpaA-GprM co-IP
54	2A_R	AGGGCCAGGGTTTTCTTCAACGTCGCCAGCCT GTTTGAGGAGAGAGAAGTTGGTGGCGCCAGAG CC	GpaA-GprM co-IP
55	Tet_gpaA_F	CTCAACTCCATCACATCACAATGGGTTGTGGA ATGAGTAC	GpaA-GprM co-IP
56	2A_GFP_R	GAAGTTGGTGGCGCCAGAGCCCTTGTACAGCT CGTCCATGC	GpaA-GprM co-IP
57	2A_gprM_F	GACGTTGAAGAAAACCCTGGCCCTATGACGAA TCGTACATCATT	GpaA-GprM co-IP
58	pYes2_troT_R	TGGCCGATTCATTAATGCAGGGCCGTAAAAAA GTTTCGGCCGGC	GpaA-GprM co-IP
59	Tet_GFP_F	CTCAACTCCATCACATCACAATGGTGAGCAAG GGCGAGGA	GpaA-GprM co-IP
