# Supplementary material for: Mitogen-Activated Protein Kinase Cross-Talk Interaction Modulates the Production of Melanins in Aspergillus fumigatus
Source: mBio. 2019 Mar 26;10(2):e00215-19. doi: 10.1128/mBio.00215-19 (PMC6437049; doi:10.1128/mBio.00215-19)
Supplement: TABLE S2 [file mBio.00215-19-st002.pdf]

Supplementary Table S2. Plasmids used in this study.

Name	Features	Usage	Reference
p123	Amp^+, *otef*p-eGFP	cloning	(6)
pCR2.1	Kan^+, Amp^+	cloning	Invitrogen
pCR2.1-*gpaA*	*gpaA* plus flanking	*gpaA* deletion	This study
pCR2.1-*gpaC*	*gpaC* plus flanking	*gpaC* deletion	This study
pCR2.1-*mpkB*	*mpkB* plus flanking	cloning	This study
pCR2.1-*mpkB*c	mpkB-*hph*	Δ*mpkB* complementation	This study
pCR2.1-*pksP*	*pksP* incomplete	*pksP* deletion	This study
pCR2.1-Δ*pksP*::*hph*	*pksP*, *hph*	*pksP* deletion	This study
pCR2.1-Δ*gpaA::ptrA*	*gpaA* flanking, *ptrA*	*gpaA* deletion	This study
pCR2.1-Δ*gpaC::ptrA*	*gpaC* flanking, *ptrA*	*gpaC* deletion	This study
pCR2.1-Δ*mpkA*::*ptrA*	*mpkA* flanking, *ptrA*	*mpkA* deletion	(7)
pCR2.1-Δ*mpkB*	*mpkB* flanking	cloning	This study
pCR2.1-Δ*mpkB*::*hph*	*mpkB* flanking, *hph*	*mpkB* deletion	This study
pCR2.1-Δ*mpkB*::*ptrA*	*mpkB* flanking, *ptrA*	*mpkB* deletion	This study
pSK275	Amp^+, *ptrA* cassette	cloning	(8)
pUC-*hph*	Amp^+, *hph* cassette	cloning	(9)
pUC-*hph*-*gpaA*	Amp^+, *hph* cassette	cloning	This study
pUC-*hph*-*gpaC*	Amp^+, *hph* cassette	cloning	This study
pYes2	Amp^+, *ura3*	cloning	Life Technologies
pYes2-*gprM*c	*gprM*-*hph*	Δ*gprM* complementation	This study
pYes2-*mpkB*-*gfp*	*mpkB* flanking, *mpkB*-*gfp*	*mpkB*-*gfp*-tagging	This study
pYes2-KM-*ptrA*	*KanMX*-*ptrA* cassette	cloning	(10)
prs426sdA::*3xHA*	*3xHA* cassette	*3xHA*-tagging	(11)
p*PTRI*	Amp^+	cloning	Clontech
pUC19*mpkA*::*3xHA*	*mpkA* flanks, *mpkA*-*3xHA*	*mpkA*-*3xHA*-tagging	This study
pYes2::*gpaA*-*gfp*	Gfp tagged *gpaA*	GpaA-GprM co-IP	This study
pTet::*gpaA*-*gfp*::*gprM*-*3xHA*	pYes2 with Tet^{on} and *ptrA*, *gpaA*-*gfp* and *gprM*-*3xHA* separated by 2A sequence	GpaA-GprM co-IP	This study
pTet::*gfp*::*gprM*-*3xHA*	pYes2 with Tet^{on} and *ptrA*, *gfp* and *gprM*-*3xHA* separated by 2A sequence	GFP-GprM co-IP	This study
